# Actin Filament Attachments for Sustained Motility *In Vitro* Are Maintained by Filament Bundling

**DOI:** 10.1371/journal.pone.0031385

**Published:** 2012-02-16

**Authors:** Xiaohua Hu, Jeffrey R. Kuhn

**Affiliations:** Department of Biological Sciences, Virginia Tech, Blacksburg, Virginia, United States of America; University of Birmingham, United Kingdom

## Abstract

We reconstructed cellular motility *in vitro* from individual proteins to investigate how actin filaments are organized at the leading edge. Using total internal reflection fluorescence microscopy of actin filaments, we tested how profilin, Arp2/3, and capping protein (CP) function together to propel thin glass nanofibers or beads coated with N-WASP WCA domains. Thin nanofibers produced wide comet tails that showed more structural variation in actin filament organization than did bead substrates. During sustained motility, physiological concentrations of Mg^2+^ generated actin filament bundles that processively attached to the nanofiber. Reduction of total Mg^2+^ abolished particle motility and actin attachment to the particle surface without affecting actin polymerization, Arp2/3 nucleation, or filament capping. Analysis of similar motility of microspheres showed that loss of filament bundling did not affect actin shell formation or symmetry breaking but eliminated sustained attachments between the comet tail and the particle surface. Addition of Mg^2+^, Lys-Lys^2+^, or fascin restored both comet tail attachment and sustained particle motility in low Mg^2+^ buffers. TIRF microscopic analysis of filaments captured by WCA-coated beads in the absence of Arp2/3, profilin, and CP showed that filament bundling by polycation or fascin addition increased barbed end capture by WCA domains. We propose a model in which CP directs barbed ends toward the leading edge and polycation-induced filament bundling sustains processive barbed end attachment to the leading edge.

## Introduction

Actin-based cell motility plays a crucial role throughout the lifetime of organism. The front or leading edge of a typical crawling cell forms a broad, fan-like lamellipodial protrusion that contains a branching actin filament network generated by the Arp2/3 complex [1], [2]. In the dendritic nucleation model of actin-based cell motility [3], [4], binding of nucleation promoting factors (NPFs), such as Wiskott–Aldrich syndrome protein (WASP) or WASP-family verprolin-homologous protein (WAVE) to the leading edge membrane exposes their active C-terminal WASP homology 2, central, and acidic (WCA) domains. Exposed WCA domains bind to an actin monomer and to the Arp2/3 complex to form a complex that binds to the side of an existing filament to generate a new, rapidly-polymerizing filament with its barbed end directed towards the membrane. The combined force of many growing actin filament barbed ends push the cell membrane outwards until each filament's growth is halted by barbed end capping protein (CP), which keeps actin filaments short and stiff. ATP-actin filaments are slowly hydrolyzed to ADP, providing a natural timing mechanism that delineates filament age and distance from the advancing membrane. Cofilin binds to and severs older ADP-actin filaments some distance away from the leading edge, and the severed oligomers rapidly depolymerize into ADP-actin monomers. Profilin replaces cofilin on actin monomers and promotes actin nucleotide exchange to provide a fresh pool of ATP-actin for filament polymerization. Profilin also suppresses *de novo* actin nucleation. In vitro these proteins are sufficient to reconstitute sustained, actin-based motility [5].

Much of the current understanding of dendritic nucleation dynamics comes from studies of *in vitro* reconstitution of actin-based motility. The pathogenic bacteria *Listeria monocytogenes* [6], [7], [8] and *Shigella flexneri* [9], [10], [11], which spread in the host body by subverting the host cell's actin motility machinery [12]. Each bacterium species expresses a single surface NPF, ActA for *Listeria* [13], [14] or IscA for *Shigella* [9], [11], which activates the cellular Arp2/3 complex to form a shell of polymerizing actin. The actin shell eventually breaks symmetry [15], [16], [17], [18] to form a branched, propulsive “comet tail” of polymerizing actin that is structurally similar to a lamellipodia [19].

Exogenous NPFs are sufficient to effect actin-based motility in cellular cytoplasmic extracts [11], [20] or in a suite of purified proteins [5]. Thus, bacteria can be substituted with microspheres [18], [21], [22], micro-discs [23], lipid droplets [24], vesicles [25], or lipid-coated particles [26], [27] that are coated with either bacterial or eukaryotic cellular NPFs such as WASP or WAVE family proteins [21], [27], [28] to study actin-based motility either in cell extracts or purified proteins.

The majority of barbed ends within a comet tail are directed toward the particle surface. How then does this filament network remain attached to the particle surface as it grows? Distortions of NPF coated lipid vesicles or droplets from round to teardrop shape show that some actin filaments must transiently attach to the particle to provide a pulling force that apposes the pushing force generated by growing barbed ends. The theoretic “tethered ratchet” model of Mogilner and Oster [29], [30] and closely related “cooperative thermal breakage” models [31], [32] predict that a subset of non-polymerizing actin barbed ends are transiently attached to the leading edge while other barbed ends push against the leading edge. The transient links are broken as the compressive force of polymerization against the barrier is translated through the crosslinked network to the attached ends. The WASP homology domain 2 (WH2, W, or V for Verprolin homology) of the cellular NPF, WASP can bind both to actin monomers [33] and the terminal subunit at the barbed end of an actin filament [34], [35], [36] and thus may provide this linkage. While some studies have shown that WH2 domains at the particle surface bind independently of the Arp2/3 complex [26], others have indirectly shown that WH2 domains maintain their attachment to filaments primarily through Arp2/3 [27] with direct WH2 to barbed end attachments playing a secondary role in a cycle of attachment, release, and elongation. While WASP likely plays roles in both maintaining comet tail attachment to the leading edge and in transiently maintaining barbed end orientation, both the mechanism and the role of Arp2/3 in this process remains unresolved.

In contrast to the tethered ratchet model, the “actoclampin” model of Dickinson and Purich [17], [37], [38] presumes that the particle surface remains processively bound to growing actin barbed ends. While processive barbed end binding has been shown for VASP [39] and formins [40], no evidence has been provided for processive attachment of WASP or WAVE proteins to barbed ends. A positively charged Arg at the N-terminus of WASP's WH2 domain alpha helix sits at the longitudinal binding region between actin subdomains 1 and 3 [36], presumably blocking further barbed end addition. However, consecutive WH2 domains such as those in neuronal Wiskott–Aldrich syndrome protein (N-WASP) have been shown to bind longitudinal actin dimers [34]. Thus, individual WH2 domains might bind to the side of filaments without steric inhibition of barbed end addition. Though processive barbed-end binding proteins such as formin and VASP are respectively dimeric and trimeric, native WASP-family proteins require binding partners such as SH3 domain or PIP_2_ at the leading edge to form multimers [41]. New evidence that the Arp2/3 complex contains two WCA binding sites [42], [43] lends support to the idea that active WH2 domains are dimeric or at high enough concentrations at the leading edge to act as multimers. As with VASP and formin, multimerization of WH2 domains might allow both bound and unbound actin subunits within the same multimer. However, processive barbed end binding by multimeric WH2 has never been reported.

As with VASP-mediated actin bundles, bundling of barbed ends at the leading edge could act synergistically to enhance the multimerization of WASP required for any processive attachment to WH2 domains. In support, several lines of evidence point to a role of actin bundling in cell motility. In an early study, melanoma cells lacking expression of the bundling protein ABP280/filamin did not migrate or produce lamellipodia compared to ABP280 expressing melanoma cells [44]. Direct perturbation of actin-fascin binding completely prevented C2C12 myoblast spreading and migration on thrombospondin-1 and partially blocked migration on fibronectin [45]. In support of the role of bundling in motility, several *in vitro* reconstitution experiments have shown that addition of filament bundling proteins increases particle propulsion rates [28], [46], [47]. Addition of filament bundling proteins α-actinin [5], T-plastin [28], or fascin [47] to a standard *in vitro* motility assay increases the propulsion velocity of tethered beads or bacteria. Once filaments are nucleated with barbed ends facing the particle, Arp2/3 appears to be dispensable to propulsion. Brieher *et al* [47] showed that *Listeria* expressing ActA on their surfaces were rapidly propelled through cytoplasmic extract by fascin-bundled filaments, even after an Arp2/3 inhibitor was added. However, the relative contribution that filament bundling plays in an Arp2/3 generated network have not been resolved.

We have used total internal reflection fluorescence (TIRF) microscopy [48], [49] and a modified *in vitro* motility assay to explore actin filament dynamics and the role of filament bundling in comet tail formation and maintenance. Thin glass nanofibers coated with WCA domains in motility buffers produced actin comet tails that propelled the particle yet were thin enough for observation of filament geometry in the comet tail. We found prominent filament bundles within the comet tail. The degree of bundle formation was controlled by CP concentration. These bundles grew faster than the surrounding dendritic actin network. Processive attachment of these fast-growing, bundled barbed ends to the particle surface often generated prominent bending and buckling of the bundle. Reduction of buffer Mg^2+^ to levels that prevented bundling without affecting actin polymerization or Arp2/3 nucleation abolished motility by eliminating comet tail formation but not shell formation or symmetry breaking. In parallel experiments with microspheres, this reduction of both persistent comet tail attachment and elongation could be rescued by addition of either excess Mg^2+^ or lys-lys^2+^. Addition of the actin bundling protein fascin rescued both comet tail attachment and elongation in low Mg^2+^, but fascin supported slower motility than did additional divalent cation. Both divalent cations and fascin promoted the direct attachment of bundled barbed ends to tethered WCA domains in a concentration dependent manner and independently of Arp2/3. We propose a model in which filament bundling allows barbed ends to cooperate for semi-processive attachment to WCA domains at the leading edge and thus help maintain the orientation of growing barbed ends at the comet tail-particle interface to generate protrusive force.

## Results

### Actin architecture in moving nanofibers

We coated thin glass nanofibers with GST-tagged WCA domains (GST-WCA) from N-WASP and observed formation of individual actin filaments with TIRF microscopy in the presence of profilin-actin, Arp2/3, and CP (Figure **S1**). We excluded cofilin, VASP, and actin bundling proteins from these experiments to understand how a minimal set of proteins direct filament organization in moving nanofibers. Although cofilin is an important player in dendritic nucleation, cofilin acts downstream of the dendritic nucleation pathway. For *in vitro* motility assays, cofilin increases propulsion rates in long-term studies by maintaining an actin monomer pool [5], [50]. We therefore restricted our initial observations to the first 30 to 90 minutes of comet tail formation. Experiments with and without cofilin showed little difference in particle velocities in similar motility buffers for these short-term observations (Table **1**).

**Table 1:** Average particle velocities in 8.5 µM profilin-actin, 100 nM Arp2/3.

Particle	[ATP]	[Cofilin]	[CP]	Tail Growth
	*mM*	µ*M*	*nM*	µ*m/min*
Nanofibers	0.38	–	75	0.08±0.05 (14)
Nanofibers	0.38	–	100	0.14±0.05 (24)
Nanofibers	0.38	–	200	0.11±0.06 (20)
4.5 µm Beads	0.38	2.0	200	0.51±0.04 (10)
4.5 µm Beads	0.38	–	200	0.48±0.12 (10)
4.5 µm Beads	0.2	–	200	0.17±0.02 (7)

In well-blocked flow-cells, nanofibers rarely adhered to the top surface but fell to the chamber bottom. We thus required a TIRF microscope with evanescent excitation at the chamber bottom to observe moving nanofibers. In contrast, previous prism-based TIRF microscopy observations of actin comet tails generated NPF-coated glass nanofibers [51] were restricted to nanofibers permanently affixed to the chamber top. Consequently, these experiments were limited to observations of actin architecture during branch initiation and not during sustained nanofiber motility.

**Figure 1:**
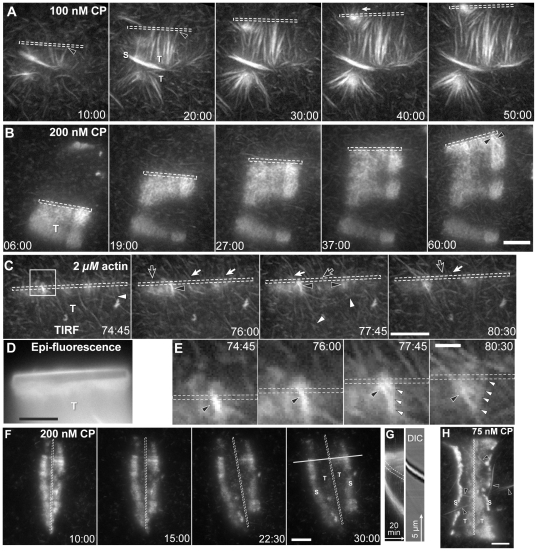
Actin filament and branch geometry in comet tails under TIRF microscopy. Conditions: A–B, 8.5 µM (8% labeled) actin, 9 µM profilin, 100 nM Arp2/3, CP as indicated; C–E, 2 µM (10% labeled) actin, 3 µM profilin, 20 nM Arp2/3, 40 nM CP; F–H, 8.5 µM (8% labeled) actin, 9 µM profilin, 100 nM Arp2/3, CP as indicated; nanofibers coated with 10 µM GST-WCA from N-WASP, motility buffer, 0.38 mM total ATP. (**A–B**) Actin architecture in comet tails (T) of moving nanofibers (dashed outline) was visible under TIRF microscopy. In 100 nM CP, comet tails consisted primarily of long filament bundles. Increasing CP to 200 nM generated a branched actin networks with short bundles (Black arrowhead). (**C**) Lowering profilin-actin, Arp2/3, and CP concentrations showed individual filaments and branches (white arrowheads) in the comet tail (T). Some filaments (white arrows) crossed the nanofiber boundary, while others terminated at the nanofiber (black arrows). Brighter filament bundles (black arrowhead) terminated at the nanofiber. (**D**) Epi-fluorescence image of panel C. (**E**) Magnified image of box in B showing bundle (black arrowhead) dissociation. The bundle was formed from daughter filaments from the same mother filament (white arrowheads). (**F**) In high CP, nanofibers sometimes formed two comet tails. (**G**) Kymograph of line in F showing tail expansion at the nanofiber surface (dashed outline) under TIRF (*left*) and DIC (*right*) microscopy. (**H**) Nanofibers sometimes formed two comet tails in low CP. Long actin bundles (black arrowheads) appeared within and beyond the comet tails. Scale bars are 1 µm for E and 5 µm for all others. Times are shown in min∶sec.

Actin shells formed around nanofibers in motility buffer at actin concentrations of 8.5 µM and a CP to Arp2/3 ratio of 0.75 to 2, often before the nanofiber drifted to the chamber bottom and into the TIRF microscopy excitation field. After initial shell formation, the nanofibers usually broke symmetry to form either one or two comet tails. Though 68% of observed nanofiber initially formed one comet tail of polymerizing actin, 32% initially formed two comet tails, one on either side of the nanofiber axis (Figure **1**F, H). In the latter case, one tail usually attained dominance and the residual tail was left behind near the original shell as the nanofiber moved (Figure **1**B). In rare cases, two tails persisted on either side of the nanofiber, and the nanofiber remained relatively stationary while the tails were pushed away from the nanofiber by new filament growth at the nanofiber surface (Figure **1**G). For both single and double comet tails, the majority of new filament growth appeared at the nanofiber surface, while the network within the comet tail either remained stationary or was pushed rearward at a slow rate. Thus, the majority of barbed end growth was directed toward rather than away from the nanofiber surface, consistent with previous reports [21], [46], [50] and the dendritic nucleation model.

Nanofibers with single tails moved at 0.14 µm/min at an optimal CP to Arp2/3 ratio of 1.0 and slightly slower at a CP to Arp2/3 ratio of 2.0 (Table **1**). These speeds were substantially slower than previous reports of 2.2 µm/min movement of spherical particles in similar buffers lacking cofilin [50]. Much of this difference could be attributed to our use of muscle actin, which produced 4-fold slower particle speeds than did cytoplasmic actin [50]. In support, we found that 4.5 µm diameter spherical particles moved at 0.48 µm/min in the same motility buffers used for nanofibers (Table **1**). These rates were comparable to previous studies in which similarly sized beads moved at 0.5 µm/min in similar motility buffers containing muscle actin [52]. Thus, the 4-fold difference between nanofiber and bead speeds in our motility buffers was due to particle geometry, and likely attributable to the thin but wide comet tails generated by 200 nm thick nanofibers.

### Capping Protein controls the transition between bundled or branched actin networks

Previous studies showed that actin filaments can form from dendritically nucleated networks *in vitro* and that CP can control the proportion of branched to bundled filaments [53], [54]. However, filament barbed ends in these studies grew away from the particle surface and bundling did not result in particle motility. We found that comet tails with barbed ends directed toward WCA-coated nanofibers also contained two populations of actin filaments. Under TIRF microscopy, bundled filaments resembling microspikes primarily formed perpendicular to the direction of movement within a dendritic network of highly branched filaments. In contrast, these bundles were invisible under epi-fluorescence microscopy (Figure **1**D). The variance in actin network architecture between bundled and branched filaments persisted as comet tails grew at the nanofiber interface. The dominance of these two structures in comet tails changed with different concentration of CP. Actin filament bundles were more prominent at lower CP concentration (100 nM) (Figure **1**A) while branched filaments dominated at higher CP concentration (200 nM) (Figure **1**B).

Actin branches and bundles were inter-convertible. Although comet tail formation was rare at lower actin concentrations of 2 µM, the origins of bundles and branches were more easily discerned in the few, sparse comet tails that formed. Here, individual filaments converged to form brighter bundles (Figures **1**E *left panel*) and bundles could sometimes be seen to dissociate into individual daughter filaments emerging from the same mother filament (Figure **1**E *right panel*).

### Bundles terminate at the nanofiber surface

At low (≤75 nM) CP concentrations, actin filament bundles frequently projected beyond the nanofiber surface as they elongated (Figure S**5**). In higher CP (≥100 nM), the majority of bundles continuously terminated at the nanofiber as they grew (Figure **1**A–C *black arrowheads*). In contrast to bundles, individual actin filaments within the comet tail often elongated across and beyond the nanofiber surface, even at higher CP concentrations (Figure **1**A, C *arrows*).

**Figure 2:**
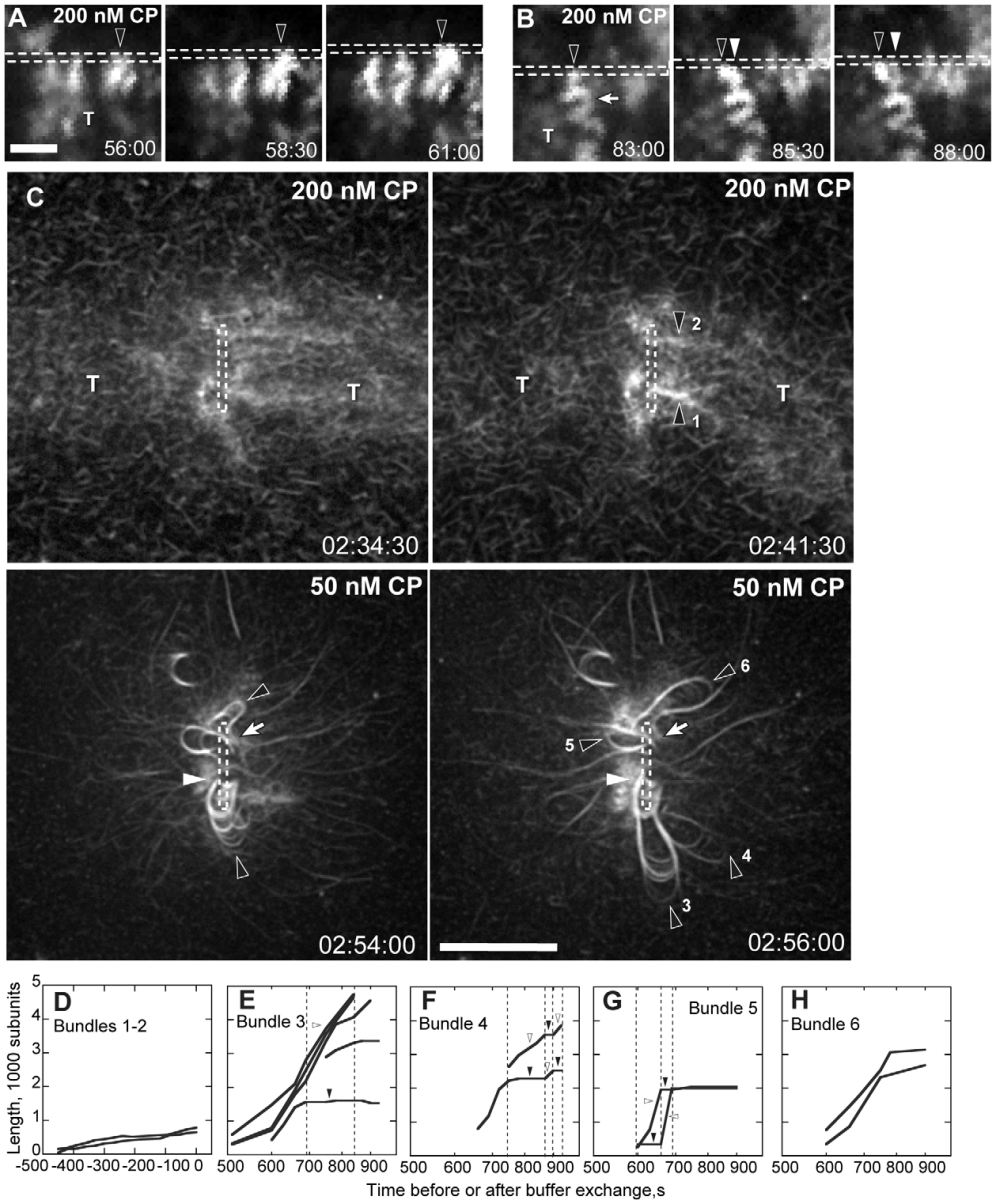
Filament bundles processively attach to the nanofiber. Conditions: 8.5 µM (8 to 20% labeled) Mg-ATP actin, 9 µM profilin, 100 nM Arp2/3, and indicated CP. Nanofiber coating and buffers as in Figure **1**. (**A–B**) Bright filament spots grew against the nanofiber surface. Spots either remained attached to the same location on the moving nanofiber (A, black arrowhead) or oscillated back and forth along the moving nanofiber (B, blacks arrowhead). Bright spots grew faster than the surrounding comet tail network (T) and often buckled from compression between the nanofiber and tail network (arrow). White arrowhead in B indicates bundle starting position. (**C**) CP was lowered from 200 to 50 nM after the establishment of two comet tails (T) by motility buffer exchange. Short, bright bundles (top panels, black arrowheads) became rapidly polymerizing bundled loops (black arrowheads, bottom panels) that remained attached to the nanofiber at their growing ends (white arrowhead) and to the tail at their pointed ends (white arrows). Growing bundles splayed into filaments of different loop lengths. (**D**) Traces of bundles lengths over time before CP reduction. Zero seconds represents the point of buffer exchange, 163 min after the experiment start. (**E–H**) Traces of individual filament lengths over time after CP reduction showed that filament within each bundle grew at different rates. Plots represent filaments from the same bundle. Some filaments continuously elongated while others show pulsed growth. Scale bars are 1 µm for A–B and 5 µm for C. Times are shown in min∶sec for A–B and hr∶min∶sec for C.

Bundles elongated faster than the surrounding comet tail, consistent with previous studies in which filament bundling proteins increased motility speeds [5], [28], [47]. For example, four bundles from Figures **2**A–B elongated at 0.22±0.05 µm/min, while the overall comet tails expanded at 0.09±0.01 µm/min. Faster bundle elongation often resulted in bending or buckling of the bundle between the fast growing barbed ends at the nanofiber surface and the pointed ends embedded within the dendritic network (Figure **2**A, B *arrowheads*).

Processive or semi-processive attachment between growing bundled barbed ends and the nanofiber surface was further illustrated by the reduction of CP concentration through an exchange of motility buffer on an existing comet tail (Figure **2**C). Here, short bundles present before the buffer exchange (top panels) elongated substantially after CP concentration was reduced (bottom panels). Processive attachment of bundled barbed ends to the nanofiber surface coupled with their faster growth rates (2.4 µm/min) resulted in significant looping of these bundled filaments. We observed similar buckling and looping of bundles on moving nanofibers (Figure S**5**, Table **2**). Thus, bundled barbed ends were not physically trapped by strong attachments between the nanofiber and the chamber surface, but were likely attached through specific molecular interactions with GST-WCA at the nanofiber surface.

**Table 2:** Frequency of bundle formation on nanofibers.

[CP]	Percentage of Nanofibers			No. Nanofibers
*nM*	with bundles	with loops	with buckles	observed
≤75	100%	27%	0%	37
100	96%	8%	25%	24
200	95%	21%	51%	57

*Conditions as in* *Table 1* *.*

As bundled loops grew, they split into individual filaments of varied lengths. Measurement of individual filament growth rates from several bundles showed that filaments within a bundle grew in a salutatory fashion (Figure **2**E–H), indicating that capping times varied between individual barbed ends within the bundle. During periods of elongation, bundled filaments grew 10-fold faster (2.4±1.1 µm/min, N = 7) after CP reduction than they did before CP reduction (0.202±0.002 µm/min, N = 2, Figure **2**D). While some filaments within a bundle elongated, others from the same bundle periodically halted. This lack of barbed end growth was likely due to capping either by CP or by WH2 domain attachment to the hydrophobic cleft of one of the barbed end subunits. In the latter case, WCA domain attachments could maintain the connection between the bundle and the nanofiber.

### Cellular levels of magnesium generate actin bundles *in vitro*

Actin bundles or microspikes form at the leading edge of motile cells *in vivo* [55] and bead-based *in vitro* motility experiments have shown that actin bundles generate more propulsive force than individual branched filaments [5], [28], [47]. Our *in vitro* experiments indicated that actin filament bundles were prevalent even in the absence of specific actin crosslinking or bundling proteins in our motility buffers. We therefore sought to eliminate bundles to determine the relative contributions of dendritic and bundled filaments to particle motility.

Both the methylcellulose used in most in vitro TIRF microscopy assays of actin dynamics [56], [57] and polyvalent cations such as Mg^2+^ [58] can bundle filaments. However, the mechanism of bundle formation by these two solutes differs substantially [59]. Methylcellulose contributes to bundle formation through entropic depletion forces that reduce effective solution volume. Polycations overcome the 14 e^−^/subunit negative charge of filamentous actin to promote filament side-to-side association.

One study of the concentration dependence of methylcellulose on bundling [60] found that filaments start to bundle in high viscosity methylcellulose (88 kDa, 4000 cPs at 2%) at a threshold concentration of 0.2%. Similar studies with polyethylene glycol showed that the filament bundling threshold also depends upon the chain length of the excluding solute [61], [62]. Although we used 0.25% methylcellulose in our TIRF microscopic assays to stabilize actin filaments for imaging, we used a lower viscosity species (63 kDa, 1500 cPs at 2%). Thus, the contribution of methylcellulose to bundling was unclear but likely near the threshold concentration.

Actin paracrystals formation requires Mg^2+^ concentrations of ≥10 mM [58], but several studies have reported anecdotal evidence that bundles form at the 1 mM Mg^2+^ concentrations used in typical TIRF actin microscopy and *in vitro* motility buffers once filament densities increase [49], [53], [63]. We therefore sought Mg^2+^ concentrations that would eliminate filament bundling in the presence of minimal, 0.25% of 63 kDa methylcellulose.

**Figure 3:**
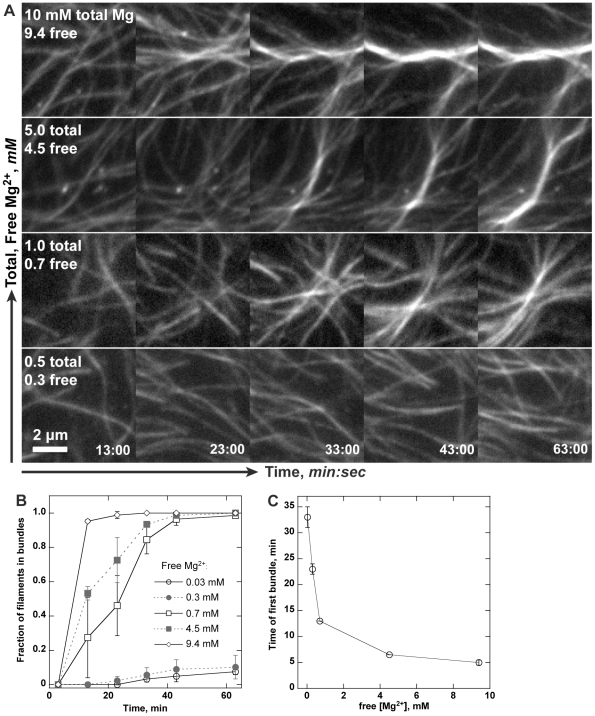
Cellular Mg^2+^ concentrations bundle actin filaments at high densities. Conditions: 2.5 µM (20% labeled) Mg-ATP actin, 50 mM KCl, 1.05 mM EGTA, 10 mM imidazole, pH 7.0, 100 mM DTT, 0.2 mM ATP, 15 mM glucose, 20 µg/ml catalase, 100 ug/ml glucose oxidase 0.25% 1500 cP methylcellulose. (**A**) Time-lapse TIRF microscopy images of *de novo* nucleated actin filaments. Images in each column were taken at the same time (min∶sec) after addition of salts. MgCl_2_ was added to low Mg-EGTA polymerization buffer to set the total Mg^2+^ as indicated. Free [Mg^2+^] was calculated from pH, ionic strength, and total Ca^2+^, Mg^2+^, EGTA, and ATP. Actin bundles readily formed at 1 mM total Mg^2+^ once filament densities increased. Increasing total Mg^2+^ to 5 or 10 mM increased the speed and extent of bundle formation. (**B**) Fraction of filaments forming bundles over time. Free Mg^2+^ concentration of at least 0.7 mM significantly increased bundle formations. (**C**) Time at which the first bundle was observed as a function of Mg^2+^ concentration. Scale bar is 2 µm.

In typical actin polymerization assays, the Ca^2+^ bound to the high affinity Mg^2+^ site on actin is first exchanged with Mg^2+^ by addition of an excess of Mg-EGTA prior to addition of KCl. To generate low Mg^2+^ conditions, we diluted Ca-ATP-actin monomers into buffer without added Ca^2+^ and reduced Mg-EGTA concentrations 5-fold during the exchange step. We correspondingly reduced ATP concentrations from 0.38 mM used above to 0.2 mM, a concentration sufficient for both actin polymerization and Arp2/3 nucleation. Total Mg^2+^ concentration in the assay was regulated by subsequent addition of MgCl_2_. We calculated free Mg^2+^ concentration from the pH and total buffer CaCl_2_, EGTA, MgCl_2_, ATP, and KCl concentrations using existing methods [64], [65]. At standard 1 mM total Mg^2+^ concentration, filament bundles could be seen after 30 min of polymerization (Figure **3**A). An increase in total Mg^2+^ to either 5 or 10 mM increased both the speed of bundle formation and thickness of bundles. Reduction of total Mg^2+^ to 0.5 mM (0.3 mM free) or below severely reduced bundle formation in the presence of 0.25% methylcellulose. Quantification of bundle formation under a range of Mg^2+^ concentrations (Figure **3**B) showed that bundles, assessed as overlap of two or more filaments, rarely formed at or below 0.5 mM Mg^2+^ but formed readily at concentration of 1 mM and above. Correspondingly, bundle onset time (Figure **3**C) was significantly shortened at high Mg^2+^ concentrations. As the typical cellular Mg^2+^ ranges from 0.5 to 1.5 mM [66], [67], [68], we expect that similar Mg-actin bundles form at the leading edge where filament density is high.

### Reduction of Mg^2+^ below cellular levels abolishes motility

**Figure 4:**
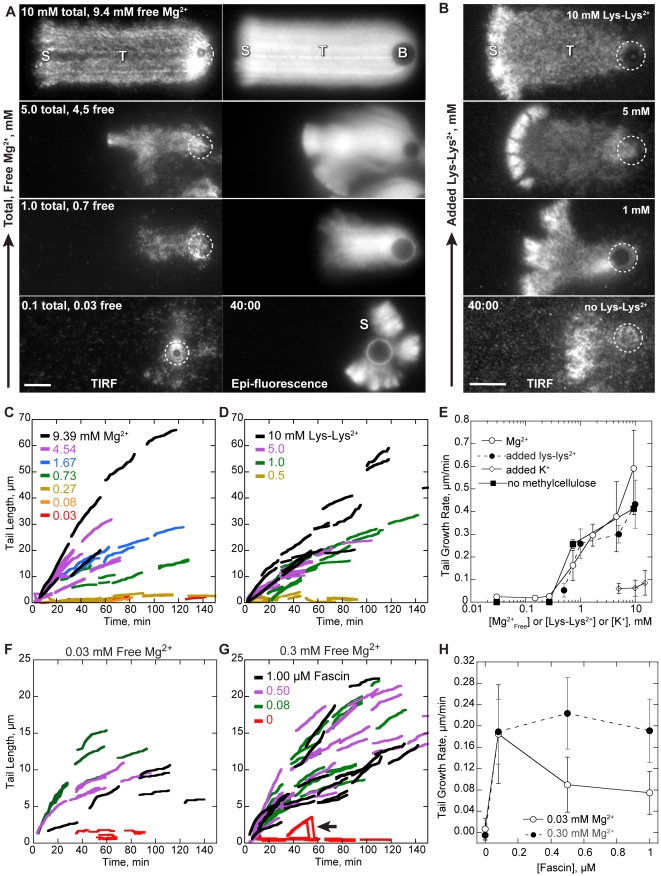
Polycations or fascin are required for bead motility. Conditions: 8.5 µM (20% labeled) Mg-ATP actin, 9 µM profilin, 100 nM Arp2/3, 200 nM CP, 4.5 µm diameter bead coated with 8.5 µM GST-WCA, low Mg^2+^ buffer (50 mM KCl, 0.105 mM MgCl_2_, 1.05 mM EGTA, 10 mM imidazole pH 7.0, 100 mM DTT, 0.2 mM ATP, 15 mM glucose, 0.25% methylcellulose, 20 µg/ml catalase, 100 ug/ml glucose oxidase) supplemented with MgCl_2_, Lys-Lys·2HCl, KCl, or fascin as indicated. (**A**) TIRF and Epi-fluorescence microscopy images show representative actin comet tails (T) grown from GST-WCA coated beads (B, dashed circle). All images were recorded 40 minutes after the reaction start. In low Mg^2+^, beads formed a shell (S) that broke symmetry but rarely a comet tail. Tails that did form remained short and detached from the bead. Restoration of cellular, 1 mM Mg^2+^ restored comet tail growth. Additional Mg^2+^ accelerated comet tail growth. (**B**) TIRF microscopy images of actin comet tails grown in 0.1 mM total Mg^2+^ with added Lys-Lys^2+^ as indicated. All images were recorded 40 minutes after the reaction start. Lys-Lys^2+^ substituted for Mg^2+^ to restore motility. (**C–D**) Comet tail growth over time after the reactions start. The lengths of actin comet tails from A–B were recorded in each frame. Line segments represent growth of individual comet tails. Comet tail growth increased with the concentration of divalent cation, either in the form of (C) Mg^2+^ or (D) Lys-Lys^2+^. (**E**) Comet tail growth rates from C–D as a function of free cation. Both MgCl_2_ and Lys-Lys-2HCl restored motility in a concentration dependent manner. Removal of methylcellulose did not influence the trend of comet tail growth rates as a function of Mg^2+^. Addition of 5, 10, or 15 mM KCl did not restore motility in low Mg^2+^ buffers. (**F–G**) Comet tail growth over time in low Mg^2+^ with added fascin. Line segments represent individual comet tails. (F) In low, 0.03 mM free Mg^2+^, 80 nM fascin optimally restored motility while (G) 500 nM fascin optimally restored motility in 0.3 mM free Mg^2+^. Line breaks (arrows) in no fascin represent growth of an actin shell followed by shell detachment during an observation. (**H**) Comet tail growth rates from F–G as a function of fascin concentration. Errors bars in E and H show S.D. of tail growth rates. Scale bars in A–B are 5 µm.

Remarkably, lowering Mg^2+^ 10-fold to 0.1 mM total (0.03 mM free) completely abolished nanofiber motility (not shown). Restoration of Mg^2+^ to 1 mM total (0.7 mM free) with additional MgCl_2_ restored nanofiber motility. While glass nanofibers provided a good model of a leading edge, they varied in length, diameter, and curvature. Thus, analysis of nanofiber motility speeds as a function of Mg^2+^ concentration could be substantially influenced by variations in nanofiber geometry. As an alternative to nanofibers, we used 4.5 µm diameter polystyrene microspheres to quantify the dependence of particle motility on Mg^2+^ concentration. We found that bead motility speeds also varied greatly with free Mg^2+^ concentration (Figure **4**A, C). Reduction of total Mg^2+^ concentration to 0.5 mM (0.3 mM free) or below abolished comet tail growth from GST-WCA coated beads.

Restoration of total Mg^2+^ to cellular concentration level of 1 mM total (0.7 mM free) restored comet tail growth rates to 0.16±0.07 µm/min (Figure **4**E). Additional of Mg^2+^ above 1 mM accelerated comet tail growth rates in a concentration-depended manner. For example, 10 mM total (9.4 mM free) Mg^2+^ accelerated comet tail growth rates to 0.59±0.17 µm/min, an approximate 4-fold increase over the rate in 1 mM Mg^2+^. Comet tail growth showed a similar dependence on Mg^2+^ in the absence of methylcellulose (Figure **4**E). These results indicate that cellular levels of Mg^2+^ are necessary for generating *in vitro* motility and that entropic depletion forces play little to no role in this requirement.

### Actin binding proteins show little Mg^2+^ dependence

Actin polymerization in KCl has been shown to be independent of excess magnesium [69]. We sought to test the possibility that Mg^2+^ was necessary for some aspect of *in vitro* motility other than actin filament bundling. We thus measured actin polymerization, Arp2/3 nucleation, and CP binding using bulk pyrene actin assembly assays in the same range of Mg^2+^ concentrations as our TIRF microscopy assays of bead motility (figure **S4**). As expected, varying free Mg^2+^ concentrations over a 300-fold range had no effect on KCl induced actin polymerization or Arp2/3 nucleation. Polymerization of profilin-actin from capped filament seeds showed a slight Mg^2+^ dependent increase in apparent final filament concentration. However, the initial slope of polymerization, an indicator of concentration of free ends, decreased slightly with Mg^2+^ concentration, and CP was the least active in low Mg^2+^ concentrations that abolished bead motility. Thus, abolishment of motility in low Mg^2+^ and restoration in high Mg^2+^ was not due to the effect of Mg^2+^ on individual components of the motility machinery.

### Cofilin does not rescue polycation-dependent motility

**Figure 5:**
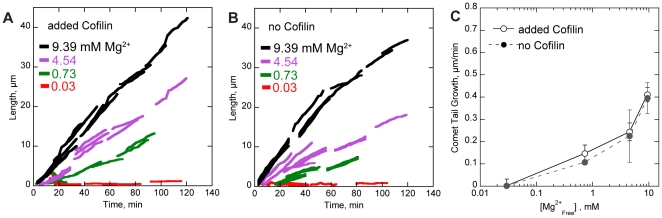
Polycation-dependent motility does not require cofilin. Conditions: 8.5 µM (20% labeled) Mg-ATP actin, 9 µM profilin, 100 nM Arp2/3, 200 nM CP, 2 µM cofilin, 4.5 µm diameter bead coated with 8.5 µM GST-WCA, low Mg^2+^ buffer as in Figure **4** supplemented with MgCl_2_ as indicated. (A–B) Comet tail growth over time after the reactions start in the presence (A) and absence (B) of cofilin. The lengths of actin comet tails were recorded in each frame. Line segments represent growth of individual comet tails. Comet tail growth increased with the concentration of Mg^2+^, either in the presence or absence of cofilin. (C) Comet tail growth rates from A–B as a function of free Mg^2+^. MgCl_2_ restored motility in a concentration-dependent manner in the presence or absence of cofilin. Errors bars show S.D. of tail growth rates.

At high Mg^2+^ concentrations, comet tail elongation slowed over the course of each 140 min experiment (Figure **4**C–D). This decrease was likely due to a decrease in available ATP-actin monomers over time from their incorporation into filaments. To test whether this decrease in monomers influenced Mg-dependent comet tail formation, we added cofilin to the motility buffer to establish a steady state of free actin monomers (Figure **5**). Cofilin addition abolished the gradual decrease in comet tail elongation over time seen at high Mg^2+^ concentrations but did not restore motility in low Mg^2+^ buffers. As with motility in buffers lacking cofilin, addition of 0.7 mM or more free Mg^2+^ restored motility in the presence of cofilin. Average comet tail growth rates were slightly higher in cofilin than without, but growth rates showed approximately the same dependence on free Mg^2+^ with or without cofilin (Figure **5**C). Coupled with the formation of both primary and secondary actin shells around beads in low Mg^2+^ buffers (see below), the lack of comet tail formation in low Mg^2+^ buffers without cofilin was not due to a lack of polymerization competent actin monomers.

### Di-lysine restores bead motility in sub-cellular concentration Mg^2+^ buffers

Based on the above observations, we reasoned that if *in vitro* bead motility depends upon the actin bundling activity of Mg^2+^ and not its coordination of ATP hydrolysis, then other polycations that bundle filaments should restore bead motility in low Mg^2+^ buffers. Oligomers of lysine have been shown by light scattering to bundle actin filaments [58], with the extent of bundling dependent upon the number of lysine residues. To match the effects of Mg^2+^ on motility as closely as possible, we added millimolar concentrations of the divalent cation Lys-Lys^2+^ to low (0.03 mM free) Mg^2+^ motility buffers. Because lys-lys^2+^ is too large to fit into the high-affinity ATP binding cleft, it would not affect Mg-ATPase activity of either Arp2/3 or actin.

Addition of 0.5 mM Lys-Lys^2+^ to low Mg^2+^ buffers did not restore bead motility, but addition of 1 to 10 mM Lys-Lys^2+^ restored bead motility in a concentration-depended manner (Figure **4**B, Figure **S2**). Although Lys-Lys^2+^ did not restore bead motility to the same extent as additional Mg^2+^ (Figure **4**D–E), Lys-Lys^2+^ is a zwitterion at cellular pH and not a true divalent cation. We expect that the negative charge of the deprotonated carboxyl group of Lys-Lys^2+^ limited the extent of its bundling activity. In contrast, addition of 5, 10, or 15 mM of monovalent KCl did not restore bead motility in low Mg^2+^ buffers (Figure **4**E). Thus, restoration of bead motility by Lys-Lys^2+^ was not simply due to an increase in buffer osmolarity or ionic strength, and *in vitro* motility absolutely requires polyvalent cations such as physiological concentrations of Mg^2+^.

### Filament bundling by fascin restores processive motility

To test if the dependence of *in vitro* motility polycations was due to actin bundling, we added the bundling protein fascin to low Mg^2+^ motility buffers and measured comet tail lengths generated by GST-WCA coated beads. In very low, 0.03 mM free Mg^2+^, addition of 80 nM fascin optimally increased comet tail growth rates (Figure **4**F, H, figure **S3**). Addition of 500 or 1000 nM fascin restored comet tail growth but to a lesser extent than did 80 nM fascin.

TIRF microscopic images of comet tails with added fascin (figure **S3**) showed a subset of straight, bright filament bundles within the comet tail that persisted throughout the entire observation period. These rigid fascin bundles were consistent with fully coupled bending in which filaments are rigidly adhered by specific crosslinks [70]. In contrast, magnesium bundles were highly dynamic and curved (Figure **2**), consistent with decoupled bundle bending and non-specific polycation-mediated adhesion between actin filaments. Thus, the failure of fascin to fully restore motility could be due to differences between fascin and magnesium bundle rigidity. In support, addition of fascin *in vitro* has been previously shown to slow initial particle motility [71], although this effect could be due to a reduction Arp2/3 mediated branching [72].

To address different contributions of fascin- and magnesium-bundled filaments to motility, we increased the free Mg^2+^ to 0.3 mM, a concentration that did not support motility on its own (Figure **4**G–H, figure **S3**). Addition of 80 nM fascin restored motility to the same extent as in 0.03 mM free Mg^2+^, but addition of 500 or 1000 nM fascin restored motility to a greater extent than in the lower Mg^2+^ concentration. Although fascin did not restore motility to the same extent as did high Mg^2+^, the difference in stiffness and persistence between fascin and magnesium bundles, coupled with competition between fascin and Mg^2+^ for inter-filament binding could explain the difference in comet tail elongation seen between fascin and high Mg^2+^.

### Filament bundling mediates sustained comet tail attachment

**Figure 6:**
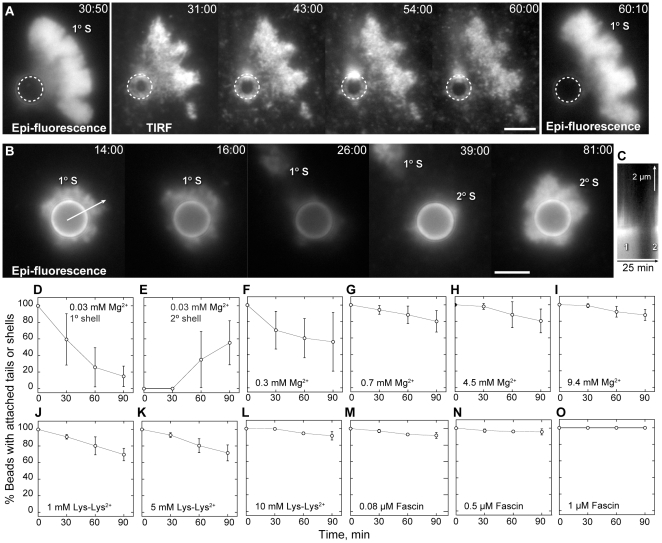
Divalent cations or fascin rescues comet tail attachment. Conditions as in Figure **4**. (**A**) Time-lapse epi-fluorescence and TIRF microscopy sequence showing detachment of primary actin shell (1°S) from GST-WCA coated bead in 0.03 free Mg^2+^. Filament density between the shell and the bead surface (dotted circle) is gradually lost. (**B**) Epi-fluorescence fluorescence microscopy showing formation of secondary actin shell (2°S) after detachment of primary actin shell (1°S) in low Mg^2+^. Times are shown as min∶sec. Scale bars are 5 µm. (**C**) Kymograph of line in B showing the detachment of primary shell (1) and establishment of a secondary shell (2). (**D–O**) Percentage of GST-WCA coated beads with either an actin shell or comet tail over time. At the reaction start, all beads developed a thin actin shell. In low 0.03 mM free Mg^2+^ buffer, actin shells detached over time (D) and many beads formed a secondary actin shell (E). Addition of 0.3 mM (F), 0.7 mM (G), 4.5 mM (H), or 9.4 mM (I) free Mg^2+^ restored shell or comet tail attachment. Addition of either 1 mM (J), 5 mM (K), or 10 mM (L) Lys-Lys^2+^ restored actin shell or tail attachment in 0.03 mM free Mg^2+^ buffer. Addition of 80 nM (M), 0.5 µM (N), or 1 µM (O) fascin restored actin shell or tail attachment in 0.03 mM free Mg^2+^ buffer. Means and S.D. were calculated from three independent experiments. At least 50 beads were counted in each experiment.

Beads in low Mg^2+^ grew thick actin shells that eventually broke symmetry (Figure **4**A, bottom panel). However, the resulting nascent comet tails did not elongate but completely detached from the bead surface and floated away (Figure **6**A). Comet tail detachment was not due to a lack of polymerization competent actin or Arp2/3, as a secondary actin shell often formed on the bead surface after the primary shell detached (Figure **6**B–C). We interpreted this comet tail detachment as a failure to establish rapid rebinding of barbed ends to WCA domain in moving particles in the absence filament bundling.

To test the dependence of attachment on bundling, we quantified the fraction of GST-WCA coated beads with attached actin shells or comet tails over time (Figure **6**D). Of the beads that initially formed actin shells in 0.03 mM Mg^2+^ buffers, these shells were eventually lost so that by 90 minutes only 15% of beads retained their primary shell and 55% of beads had formed secondary shells (Figure **6**E). Additional of Mg^2+^ restored bead-actin attachment in a concentration dependent manner (Figure **6**F–I). Increased comet tail growth rate was correlated with increased comet tail attachment. Therefore, Mg^2+^ induced bundling was likely required to maintain comet tail attachment to WCA at the bead surface to sustain bead motility. Similarly, addition of lys-lys^2+^ (Figure **6**J–L) or fascin (Figure **6**M–O) also restored sustained attachment of comet tails to the bead surface in 0.03 mM free Mg^2+^ buffers in a concentration dependent manner. Restoration of attachment was most dramatic in 1 µM fascin, as 100% of beads retained their comet tails over 90 minutes of observation. Thus, filament bundling did not mediate actin shell formation but continued attachment between actin filaments and the bead surface once motility was established.

### Bundling enhances barbed end binding to WCA domains in the absence of Arp2/3 and CP

**Figure 7:**
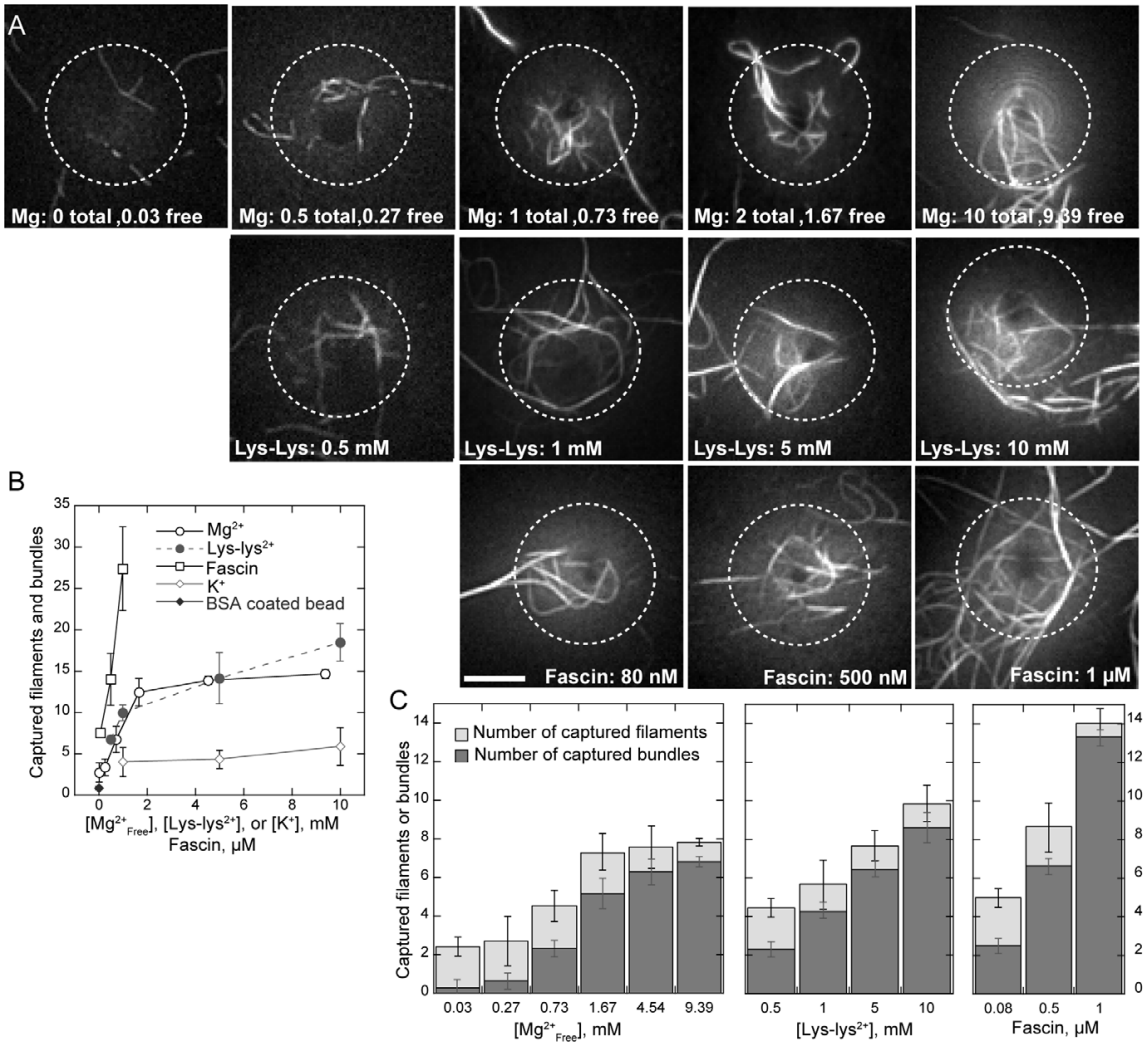
Bundling promotes barbed end attachment to WCA domains in the absence of Arp2/3 complex. Conditions: 8 µM (30% labeled) actin was polymerized in low Mg^2+^ F buffer. Filaments were incubated with GST-WCA coated microspheres with 1 µM ATP-actin monomers and the indicated final concentration of Mg^2+^, Lys-Lys^2+^, fascin, or K^+^ for 10 minutes. Beads were centrifuged, resuspended in low Mg^2+^ buffer in the absence of methylcellulose, and imaged on poly-lysine coated coverslips. (**A**) TIRF microscopy images of actin filaments and bundles attached to the bottom of coated microspheres in the indicated concentration of Mg^2+^, Lys-Lys^2+^, fascin, or K^+^. The number of actin filaments and bundles crossing the bead boundary (dashed circle) were counted for each bead. Scale bar is 2 µm. An example measurement is shown in Figure S**6**. (**B**) Count of average number of captured filaments per bead as a function of Mg^2+^ (○), Lys-Lys^2+^ (•), fascin (□), or K^+^ (⋄) concentration. Bundles were counted as two filaments. Error bars show S.D. from at least 60 beads for each condition from three independent experiments. Coincidental filament overlap with control, BSA coated microsphere (⧫) was negligible. (C) Stacked bar chart showing average number of filaments (light gray) or bundles (dark gray) captured by WCA-coated microspheres, with indicated Mg^2+^, Lys-Lys^2+^, fascin concentrations. The proportion of captured bundles increased with increasing polycation or fascin.

To better dissect the role of filament bundling played in barbed ends binding to leading edge, we captured pre-formed filaments by GST-WCA coated beads in the absence of profilin, Arp2/3, and CP. Fluorescent actin was polymerized in low Mg^2+^ buffer and sheared to increase the number of ends. Short filaments were then incubated for 10 minutes with GST-WCA coated beads, actin monomers in low Mg^2+^ buffer, and added Mg^2+^, Lys-Lys^2+^, or fascin. After the beads were separated by centrifugation, filaments captured along the bottom surface of the bead were imaged by TIRF microscopy (Figure **7**A). We scored filaments or bundles that terminated at the bead surface as captured barbed ends (Figure S**6**). Although we could readily discern individual filaments from bundles under TIRF microscopy, we could not accurately determine the number of filaments within each bundle. We therefore scored bundles as having two barbed ends for the purpose of estimating the total number of captured ends. Counts of total filament ends captured per bead (Figure **7**B) showed that additional Mg^2+^, Lys-Lys^2+^, or fascin increased barbed end capture by tethered GST-WCA. Barbed end capture in low Mg^2+^ was only slightly higher than the number of filaments that coincidentally overlapped with control bovine serum albumin (BSA) coated beads (0.9±0.2).

Among all three bundling factors, 1 µM fascin provided the highest number of captured barbed ends (27±5 per bead), a 10-fold increase over the number of barbed ends captured in 0.03 mM free Mg^2+^ (2.7±1.2 per bead). We note that while tail speeds in 0.03 mM Mg^2^ decreased at higher fascin concentrations (Figure **4**H), fascin addition in 0.03 mM Mg^2+^ monotonically both increased actin filament capture (Figure **7**B) and prevented comet tail detachment (Figure **6**M–O). Thus, the decrease in minimal Mg^2+^ motility rates in higher fascin shown in Figure **4** was likely due to fascin-induced differences in filament geometry or elongation within the comet tail and not due to failure of barbed end attachments to the bead surface at higher fascin levels.

Increased barbed end capture by increased Mg^2+^ was not due to an increase in the number of filaments available for capture as the Mg^2+^ range used did not affect actin polymerization (Figure **S4**A), nor was increased capture due solely to increased ionic strength as additional KCl only minimally increased the number of captured ends (Figure **7**B). Further analysis of captured barbed ends (Figure **7**C) showed that the number of single filaments captured did not vary substantially across all conditions. Beads captured from 2 to 1 individual filaments with increasing concentrations of Mg^2+^ or Lys-Lys^2+^ and from 2.5 to 0.7 individual filaments with increasing fascin. Rather, the increase in captured barbed ends resulted from a substantial increase in the number of captured bundles. Thus, capture of barbed ends by tethered WCA domains was largely due to filament bundling and WCA binding did not require Arp2/3 or CP.

## Discussion

We used TIRF microscopy to observe the generation and maintenance of actin filament architecture in an *in vitro* motility system that utilized thin glass nanofibers as an analog for the leading-edge membrane of a motile cell. Our assay differed from previous TIRF observations of nanofiber-supported nucleation [51] in that our nanofibers moved within the TIRF excitation field rather than being immobilized. Thus, we were able to observe actin filament architecture during the later stages of sustained nanofiber motility after shell formation and symmetry breaking.

Our central finding was that filament bundling was essential for maintaining persistent attachments between growing barbed ends and N-WASP WCA domains on the moving particle. Multiple lines of evidence support this claim. (1) Bundles form within the dendritic network in the absence of bundling proteins. While entropic depletion forces contribute to bundling, bundling is primarily due to cellular concentrations of the polyvalent cation, Mg^2+^. (2) CP antagonizes bundle length, but short bundles are still generated within the comet tail even at high CP concentrations. (3) Like branched filaments, bundled barbed ends face the particle surface and can bind to WCA domains independently of Arp2/3. In moving nanofibers, these barbed end attachments are semi-processive and provide enough force to cause significant buckling of short bundles or looping of longer bundles. (4) Although filament bundling does not affect actin shell formation or symmetry breaking, bundling is required to maintain continued attachments between the growing comet tail and the particle surface. (5) WCA-tethered bundles elongate faster than the surrounding dendritic network, suggesting that WCA binding to bundled barbed ends antagonizes CP binding.

It is not surprising that bundles form in the absence of bundling proteins. In published ultrastructure studies of dense actin branched actin in motile cells [2], [73], the leading edge contains a wealth of short, parallel actin filaments consistent with polycation-mediated bundles. Although many of the longer bundles are generated by VASP, VASP remains at the elongating bundle tip and fascin recruitment to the bundle is delayed [74]. Polycations-mediated bundling could serve as the bridge between initial bundle formation and bundle stabilization by specific bundling proteins. We propose that filament densities at the leading edge are high enough that filaments likely form initial bundles when cellular Mg^2+^ concentrations exceed 0.5 mM. Other cellular polycations such as spermine may further promote short filament side-to-side association typically seen in nascent filopodia or microspikes. Thus, multivalent cation-induced filament bundling in the absence of specific bundling proteins may be more important to dendritic nucleation than previously thought.

**Figure 8:**
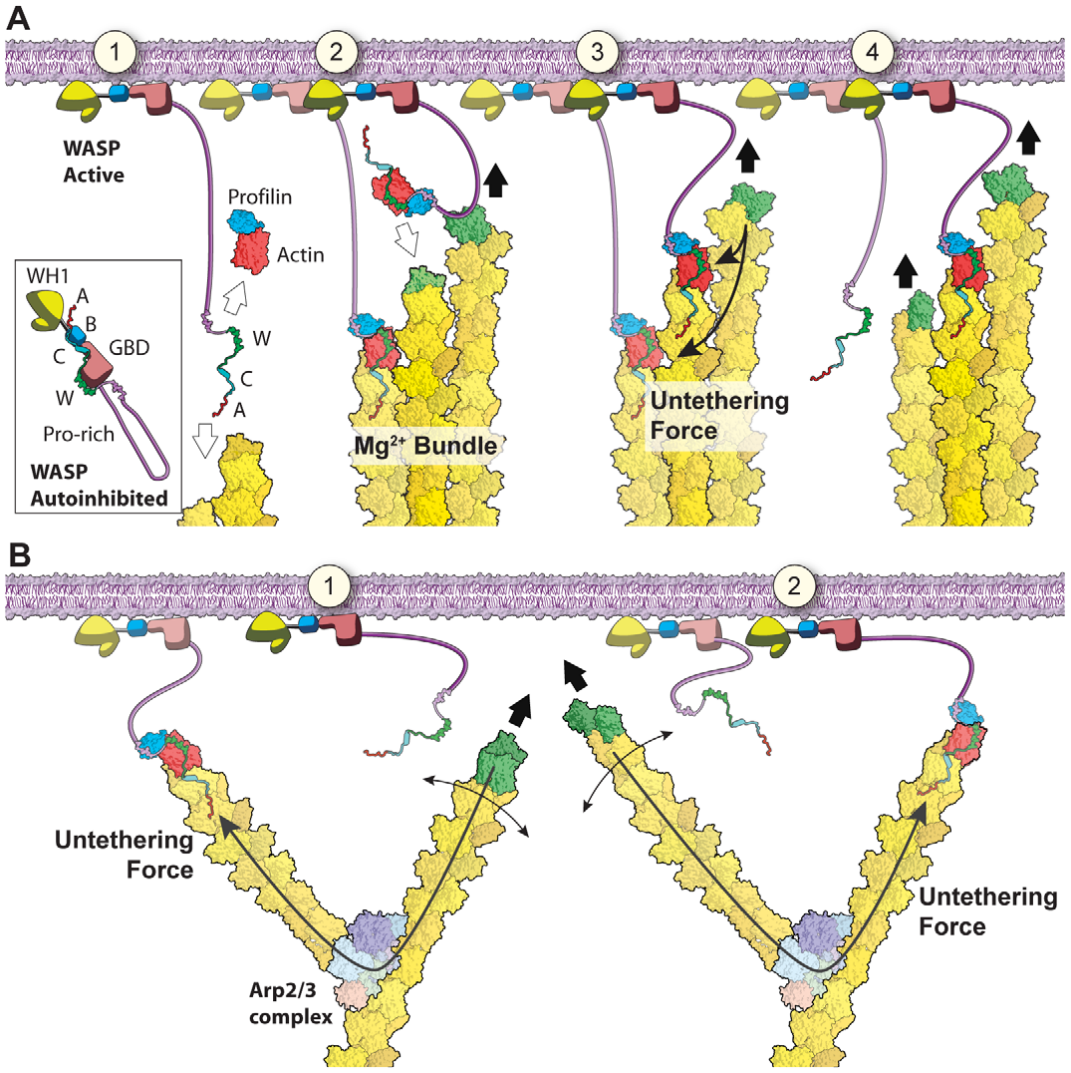
Model of bundle and branch cooperativity. (**A**) Bundles cooperate efficiently to maintain barbed end orientation. (1) WASP binding to membrane and Rho family GTPase frees active WCA domains that can either bind to a free profilin-actin or the barbed end of a nearby filament bundle. (2) One filament in a bundle is attached to WCA (red), while sister filaments are free to either polymerize by subunit addition (green), bind to a nearby free WCA, or (3) bind to a profilin-actin bound WCA. The force of polymerization is efficiently transmitted through the stiff bundle to tethered barbed ends and promotes tether dissociation. (4) Dissociation of bound WCA frees a sister barbed end for polymerization. (**B**) Branches cooperative inefficiently to maintain barbed end orientation. (1) While one barbed end is tethered to the membrane through WCA, a nearby barbed end polymerizes against the membrane (green). The force of polymerization is transmitted through branches and flexible filaments to tethered barbed ends to promote tether dissociation. (2) WCA dissociation frees a barbed end for polymerization while other filaments within the branched network become tethered.

How might actin bundling promote processive barbed end attachment to monomeric WH2 domains at the leading edge? Both formin dimers [40], [75] and VASP tetramers [39], [76] can remain processively attached to growing barbed ends. Although WASP family proteins are monomeric in solution, they are present at high concentrations at the leading edge membrane and multiple WCA domains from adjacent WASP family proteins could cooperate to attach to filament barbed ends (Figure **8**A). Bundling of filaments by divalent cations aids WCA binding to barbed ends at the leading edge (Figure **8**A). Bundling would switch barbed end WCA attachments from slow, “cooperative thermal breakage” attachments (Figure **8**B) found in branched networks [31], [32] to fast, processive attachments (Figure **8**A) to WCA domains. Dimerization of WASP/WAVE has been shown to greatly enhance Arp2/3 activation activity [41], [77]. Similarly, dimerization of WASP/WAVE family WCA domains at the leading edge could act synergistically with filament bundling to enhance processive binding to barbed ends. In support, we note that our study used GST-tagged WCA, which has been shown to form dimers *in vitro* [41].

Although the affinity of WCA domains for actin monomers is weak (K_d_ of 0.6 µM [33]), bundled filament barbed ends could cooperate to maintain processive attachments to several nearby WCA domains at the nanofiber surface. As one barbed end binds to a WCA domain, sister barbed ends within the bundle would be free to elongate. Rapid transmission of compressive force from free barbed ends polymerizing against the barrier to WCA-attached barbed ends within the same bundle would accelerate their detachment. In this way, the filament bundle could processively elongate and remain attached although individual ends would be free to attach and detach from WCA domains during polymerization.

Like VASP, cooperative enhancement of the apparent WCA-barbed end affinity might also allow several nearby WCA domains to compete with the high affinity binding of CP to barbed ends (K_d_ of ∼0.1 nM [78]), either by delaying CP association or accelerating CP dissociation at the nanofiber surface. Decreased CP activity near the NPF-coated surface would bias productive barbed end elongation towards the surface, while nonproductive Arp2/3 nucleated barbed ends that were pointed away from the surface would be rapidly capped.

Though we showed that motility *in vitro* requires polycations, the requirement of polyvalent cations such as magnesium for motility in vivo remains unclear. Magnesium has long been associated with integrin-mediated cell attachments that provide traction for moving cells [79]. The metal ion-dependent adhesion site (MIDAS) extracellular domains found in all β- and some α-integrins requires Mg^2+^ to coordinate extracellular matrix binding [80], [81], [82], [83]. For example, wound healing *in vivo* is blocked by chronic removal of Mg^2+^ from the wound fluid [84]. However, cell spreading is also an actin polymerization-driven process very similar to directed motility [85], [86], [87], [88]. Like Ca^2+^, Mg^2+^ levels can be acutely increased by release from mitochondrial, nuclear, and ER stores, and chronic exposure of cells to low extracellular Mg^2+^ would deplete stores.

Could the requirement of extracellular Mg^2+^ for integrin-mediated cell adhesion have masked its intracellular requirement for actin-based motility? Typical cellular free Mg^2+^ concentrations range from 0.5 to 1.5 mM [66], [67], [68], [89], precisely the range at which we find the greatest effect on processive motility in vitro (Figure **4**). While it should be noted that cellular Mg^2+^ measurements have varied for the same cell type [89], intracellular Mg^2+^ levels have been shown to increase upon cell stimulation. For example, free cellular Mg^2+^ levels in platelets increase from 0.6 to 1.27 mM upon insulin stimulation [90] and from 0.5 to 1.3 mM upon thrombin stimulation [91].

High cellular Mg^2+^ levels have been linked to angiogenesis and endothelial cell migration. Though Mg^2+^ increases cell proliferation [92], its main action during angiogenesis appears to be linked to motility [93]. Lapidos et al [94] found that Mg^2+^ acted as potent chemoattractant for endothelial cells. Chemotaxis towards Mg^2+^ was blocked by inhibition of G_α_i heterotrimeric G-proteins. Thus, Mg^2+^ chemotaxis involved second messengers and was not due solely to a gradient of integrin-mediated adhesion. Studies of free cytoplasmic Mg^2+^ with mag-fura-2 showed that cytoplasmic Mg^2+^ rapidly increased in endothelial cells from basal levels of 0.5 mM to 1.1–1.2 mM upon stimulation with the chemoattractants VEGF [95] or bFGF [96] due to release from intracellular stores. While this study does not directly address cellular Mg^2+^ levels, our findings point to direct participation of Mg^2+^ in the motility process.

## Materials and Methods

### Protein expression, purification, and fluorescent labeling

Actin was purified from rabbit skeletal muscle acetone powder through one round of polymerization, depolymerization, and gel filtration [97]. Actin was labeled with pyrenyl iodoacetamide (Invitrogen, Carlsbad, CA) [98] or with Oregon green 488 iodoacetamide (Invitrogen) as previously described [49]. Before use, labeled and unlabeled actins were dialyzed overnight against fresh buffer G (2 mM Tris-Cl pH 8, 0.2 mM ATP, 1 mM NaN_3_, 0.1 mM CaCl_2_, 0.5 mM dithiothreitol, DTT) and centrifuged at 100,000 g for 2 hr at 4°C. Arp2/3 complex was purified from bovine thymus as described [99]. Recombinant mouse capping protein was expressed in *E. Coli* and purified as described [100]. Human N-WASP-WCA was expressed as a glutathione S-transferase (GST) fusion proteins in *E. Coli* and purified on a Glutathione Agarose affinity column (Thermo Scientific, Rockford, IL) followed by anion exchange chromatography on a Source Q (GE Healthcare, Piscataway, NJ) column [33]. Recombinant human profilin I was expressed in *E. Coli* and purified by poly-L-proline affinity chromatography as described [101]. Recombinant human fascin I was expressed in *E. Coli* and purified and cleaved as described [102]. Actin and labeled actins were stored for 1 month at 4°C. All other proteins were flash frozen in liquid nitrogen and stored at −80°C.

### Nanofiber and bead preparation

Nanofibers (200 nm nominal diameter, Johns Mansville, Denver, CO) were separated in chloroform with a Dounce homogenizer, centrifuged at 3750 rpm for 15 min in a clinical centrifuge, and the chloroform was evaporated in a fume hood. Nanofibers were washed once with deionized water by centrifugation and sonicated for 1 hour in 1 M KOH in a bath sonicator to remove contaminants. Nanofibers were washed briefly in deionized water, resuspended in 1 M HCl, sonicated for 1 hour, and incubated overnight in HCl. Cleaned nanofibers were subsequently pelleted by centrifugation and sonicated for 30 minutes each in in ddH_2_O, 1 mM ethylene diamine tetraacetic acid (EDTA), 70% ethanol, and absolute ethanol to dry, with pelleting between each step. Cleaned nanofibers were stored in glass containers in absolute ethanol for up to three months.

Carboylated polystyrene 4.5 µm diameter microsphere (Polysciences, Warrington, PA) or glass nanofiber were coated respectively with 8.5 uM or 10 uM GST tagged WCA by incubation for 1 h at room temperature. Particles were pelleted by low speed centrifugation and resuspended in storage buffer (10 mM HEPES pH 7.8, 0.1 M KCl, 1 mM MgCl_2_, 1 mM ATP, 0.1 mM CaCl_2_, 0.01% NaN_3_) containing 1 mg/ml bovine serum albumin (BSA, Sigma-Aldrich, St. Louis, MO) to block subsequent nonspecific binding. Particles were stored at 4°C for up to 1 week.

### Reconstitution of nanofiber and bead motility under TIRF microscopy

Glass slides and coverslips were cleaned, and flow cells constructed as previously described [49]. For buffer exchange, flow cells were coated with 300 nM n-ethylmaleimide inactivated myosin II for 2 minutes. For all experiments, flow cells were coated with 1% BSA for 5–7 min as described [49]. After blocking, 16 µl of reaction mixture was wicked through the chamber and the chamber was either sealed with warm VALAP (1∶1∶1 vaseline/lanolin/paraffin) or left unsealed for subsequent buffer exchange. For bead motility assays, glass slides and coverslips were blocked overnight in 1% BSA at 4°C and dried in air before use. We placed 16 µl of reaction mixture on a BSA coated slide, covered with a BSA coated coverslip, and sealed the chamber with VALAP.

Labeled and unlabeled Ca-ATP actin were diluted to the desired labeled fraction, mixed 9∶1 with 10× magnesium exchange buffer (10× ME: 10 mM ethylene glycol tetraacetic acid, EGTA, 1 mM MgCl_2_) and incubated on ice for 2 minutes to form 4× final concentrations of Mg-ATP actin. We placed 8 µl of Mg-ATP actin at the bottom of a 1.5 ml Eppendorf tube and added 7 µl of motility protein mixtures and 1 µl of coated nanofibers or beads on the side of the tube. Arp2/3 and CP were diluted in nanoparticle storage buffer (10 mM HEPES pH 7.8, 0.1 M KCl, 1 mM MgCl_2_, 1 mM ATP, 0.1 mM CaCl_2_, 0.01% NaN_3_). We washed both drops together with 16 µl 2× TIRF buffer (2×: 100 mM KCl, 2 mM MgCl_2_, 2 mM EGTA, 20 mM imidazole, pH 7.0, 200 mM DTT, 0.4 mM ATP, 30 mM glucose, 0.5% 1500 cP methylcellulose, 40 µg/ml catalase, 200 ug/ml glucose oxidase) and placed the reaction mixture in either a flow cell or slide-coverslip as described above.

### Image acquisition and processing

Actin fluorescence was observed with a 60×1.49 NA TIRF objective on an Olympus IX2 inverted microscope. Images were captured with a Retiga EXi cooled CCD camera (QImaging) using SlideBook image acquisition software (Intelligent Imaging Innovations, Inc). All subsequent image-processing steps were performed in ImageJ, available at http://rsbweb.nih.gov/ij. TIRF microscopy images were gamma corrected using a value of between 0.5 and 0.8. An unsharp-mask filter was applied with a radius of 1 to 1.3 pixels and a 60% mask weight. epi-fluorescence microscopy images were unprocessed. For differential interference contrast (DIC) images, each image was divided by an averaged background image and contrast was adjusted to locate the nanofiber. Images were rotated and cropped for publication.

### Bundle formation during nanofiber motility

To quantify the frequency of bundling, loop, and buckle formation (Table **2**), we counted the number of nanofibers with obvious filament bundles and divided by the total number of nanofibers observed. For nanofibers with bundles, we classified those bundles as looped, buckled, or neither. Looped or buckled bundles exhibited significant curvature and had one end terminating at the nanofiber surface and the other end embedded in the comet tail. Looped bundles were longer and had one significant curve. Buckled bundles were shorter and had two or more curved sections.

### Bead motility in low magnesium

For low Mg^2+^ motility buffers, we made the following changes: (1) For each experiment, we diluted fresh Ca-ATP actin into buffer G with no added Ca^2+^. (2) We mixed actin 9∶1 with 10× low magnesium exchange buffer (10× lowME: 2 mM EGTA, 0.2 mM MgCl_2_) for 2 minutes to form 4× final concentration of actin. (3) We diluted all proteins in buffer G with no added Ca^2+^, or Mg^2+^, or EGTA. (4) We reduced MgCl_2_ in 2× TIRF buffer from 2 mM to 0.2 mM to form 2× low-ME TIRF buffer. The final total concentration of Mg^2+^, Ca^2+^, EGTA, and ATP were 0.105 mM, 5 µM, 1.05 mM, and 0.2 mM, respectively. Free Mg^2+^ and Ca^2+^ concentrations were calculated using MaxChelator software [64], [65] available from maxchelator.stanford.edu.

### Comet tail detachment

To quantify detachment of comet tails or actin shells from beads, we placed GST-WCA coated microspheres in low Mg^2+^ motility buffers as above and counted number of beads with comet tail or actin shell under epi-fluorescence microscopy every 30 minutes. We divided this count by the total number of beads observed per experiment. Actin shells were defined as a circumferential increase in actin polymerization around the bead surface. Shell detachment was scored as an absence of actin fluorescence in a ∼1 µm zone around the bead circumference. Detachment was often accompanied by a nearby empty shell or nascent comet tail. Average and S.D. of for each condition were calculated from three independent experiments. At least 50 beads were scored for each experiment.

### Filament bundling in low and high magnesium

Labeled and unlabeled Mg-ATP-actin were diluted in buffer G without added calcium as above, mixed with low Mg^2+^ motility buffer supplemented with MgCl_2_, added to chambers pre-blocked with 1% BSA, and filament growth was observed by TIRF microscopy. To quantify bundling, a 47 µm×35 µm subfield at the center of each video was chosen and the total numbers of individual filaments and the number of filaments converging into bundles were scored. Bundles were scored as filaments overlapping for two or more frames. The fraction of bundled filaments was scored from three independent experiments for each condition.

### Filaments binding to microspheres

Coverslips were coated with 1 mg/ml poly-L-lysine (30 to 70 kDa, Sigma-Aldrich) for 2 minutes, rinsed 3× with deionized water, and dried in air. We polymerized 8 µM (30% Oregon green labeled) Mg-ATP actin in buffer F (buffer G with 10 mM Imidazole pH 7, 50 mM KCl, 0.105 mM MgCl_2_, 1 mM EGTA) for 10 minutes at room temperature. Actin seeds were vortexed at maximum speed for 1 minute to break filaments. To prepared seeds, we added 4.5 µm diameter microspheres coated with 8.5 µM GST-WCA, 1 µM Oregon green labeled Mg-ATP actin monomers, and 1 mg/ml BSA. Mg^2+^, Lys-Lys^2+^, fascin or K^+^ were also added at this step. The reaction was incubated for 10 minutes at room temperature, beads and bound filaments and bundles were pelleted by centrifugation at 5000 rpm for 1 min, and the supernatant was removed. The pellet containing beads and bound filaments was gently diluted 16-fold in buffer F, mixed 1∶1 with 2× TIRF buffer in the absence of methylcellulose, and 16 µl was added to a poly-L-lysine coated coverslip. Filaments/bundles and beads were observed, respectively, by TIRF or DIC microscopy.

### Bulk pyrene-actin spectroscopy assays

For actin and profilin-actin experiments, we diluted labeled and unlabeled Ca-ATP actin to 30% labeled fraction with or without added profilin, mixed 9∶1 with 10× low-Mg exchange buffer, and incubated on ice for 2 minutes to form 2× final concentrations of Mg-ATP actin in the lower row (preparatory wells) of a 96 well half area flat bottom plate (Corning). Various concentrations of MgCl_2_ were placed in the upper row (reaction wells) of the same plate along with 1.6 µl of 100× antifoam (100×: 0.005% antifoam-204, Sigma-Aldrich), 2× initial concentration of low-Mg KMEI (10×: 500 mM KCl, 1 mM MgCl_2_, 10 mM EGTA, 100 mM Imidazole, pH 7), and buffer G without added Ca^2+^. We started the reaction by transferring 80 µl of actin mixture from the lower preparatory row to the upper reaction row containing 80 µl in each well for a 160 µl total reaction. The reaction was gently mixed with a 12-channel pipette, and pyrene-actin fluorescence was measured in a Spectra MAX Gemini XPS fluorescent plate reader (Molecular Devices, Sunnyvale, CA) with excitation and emission wavelengths of 364 nm and 407 nm, respectively. Arp2/3 nucleation experiments were similarly performed with Arp2/3 and GST-WCA added to the reaction well prior to the reaction start. For capping protein activity assays, we added CP to the reaction well followed by short (vortexed) unlabeled actin filament seeds prior to the reaction start as previously described [103].

## Supporting Information

Figure S1 **Coomassie stained SDS-PAGE gel of purified motility proteins.** Lane 1, unlabeled rabbit skeletal muscle actin; Lane 2, Oregon green 488 labeled skeletal muscle actin; Lane 3, bovine thymus Arp2/3 complex; Lane 4, recombinant human profilin; Lane 5, recombinant mouse capping protein; Lane 6, recombinant glutathione sepharose transferase (GST) N-terminal tagged WCA domains from human N-WASP; Lane 7, recombinant human fascin; Lane 8; rabbit skeletal muscle myosin II heavy chain inactivated with N-Ethylmaleimide.
(TIF)

Figure S2 **Lys-Lys2+ restores motility.** Conditions as in Figure **4**B. TIRF and epi-fluorescence microscopy images of actin shells (S) and comet tails (T) grown from GST-WCA coated beads in 0.1 mM total, 0.03 mM free Mg^2+^ buffer with added Lys-Lys^2+^ as indicated. Each image was recorded 40 minutes after initiation of the reaction. Lys-Lys^2+^ substituted for Mg^2+^ to restore motility. Scale bar is 5 µm.
(TIF)

Figure S3 **Fascin restores motility.** Conditions as in Figure **4**. (**A**) TIRF and epi-fluorescence microscopy images of actin shells (S) and comet tails (T) grown from GST-WCA coated beads in 0.1 mM total, 0.03 mM free Mg^2+^ buffer with added fascin as indicated. Fascin added to 80 nM optimally restored comet tail elongation. Straight fascin bundles (black arrowheads) can be seen both within the comet tail and in the surrounding media. (**B**) Actin shells (S) and comet tails (T) grown in 0.5 mM total, 0.3 mM free Mg^2+^ buffer with added fascin as indicated. Although 0.3 mM free Mg^2+^ did not support motility on its own, fascin addition restored motility to a greater extent than in 0.03 mM free Mg^2+^. Each image was recorded 40 minutes after initiation of the reaction. Scale bar is 5 µm.
(TIF)

Figure S4 **Minimal Mg^2+^ is sufficient for actin polymerization, Arp2/3 nucleation, and CP activity.** Polymerization of pyrene actin in low Mg^2+^ buffer (50 mM KCl, 0.105 mM MgCl_2_, 1.05 mM EGTA, 10 mM imidazole pH 7.0, 0.2 mM ATP). MgCl_2_ was added to generate indicated free [Mg^2+^]. (**A**) Polymerization of 8.5 µM (30% pyrene labeled) Mg-ATP-actin induced by KCl was not affected by MgCl_2_ concentration. (**B**) Addition of 8.5 µM human profilin to 8.5 µM actin did not affect Mg^2+^ independent actin polymerization. (**C**) Nucleation of 2 µM (30% labeled) Mg-ATP-actin by 40 nM Arp2/3 and 500 nM bovine N-WASP WCA. Mg^2+^ did not affect the time course or extent of Arp2/3 mediated nucleation. (**D**) Nucleation conditions in C with addition of 2 µM profilin. Profilin did not significantly alter the Mg^2+^ independence of Arp2/3 nucleation. (**E**) Polymerization from capped seeds. Short unlabeled actin seeds diluted to 1.2 µM filament were incubated with 0.2 nM CP or buffer alone (no CP). Capped seeds were added to 1 µM (30% pyrene labeled) actin, 3 µM profilin at the reaction start. (**F**) Normalized initial slope from the first 200 s of polymerization from capped seeds in E.
(TIF)

Figure S5 **Looped bundles formed in low CP.** Conditions: 8.5 µM (8% labeled) actin, 9 µM profilin, 100 nM Arp2/3, CP as indicated. (**A–C**) At low CP concentrations, bundled loops (*black arrowheads*) often formed on both stationary (A) and moving (B–C) nanofibers. Loops grew with one end embedded in the comet tail (T) and the other attached to the nanofiber surface (*dashed outline*). In additional to looped bundles, straight bundles (*white arrows*) often projected beyond the nanofiber surface at low CP concentrations. Scale bar, 5 µm. Time, min: sec.
(TIF)

Figure S6 **Scoring of bundles captured by GST-WCA coated beads.** (A) Sample TIRF microscopy image of actin filaments captured by a GST-WCA coated bead (*dashed circle*) in 0.03 mM free Mg^2+^ supplemented with 1 mM Lys-Lys^2+^. Scale bar, 5 µm. (B) Profile plot of fluorescent intensity along the line in A that intersects seven filaments. Numbered peaks correspond to marked filaments. Two dim background filaments are included for comparison. (C) Sample scoring method for experiments shown in Figure **7**. Camera gain, acquisition time, and display range were kept constant between experiments to give roughly the same apparent magnitude (peak-to-trough intensity) of background filaments (6–7). Filaments were scored as captured (+) if they crossed or were contained within the bead boundary as measured with DIC microscopy. Captured filaments with apparent magnitudes similar to background filaments were scored as individual filaments (Fil). Captured filaments with apparent magnitudes of at least double the average magnitude of background filaments were scored as bundles (Bun).
(TIF)
