# Supplementary figures and images for: Actin Filament Attachments for Sustained Motility In Vitro Are Maintained by Filament Bundling
Source: PLoS One. 2012 Feb 16;7(2):e31385. doi: 10.1371/journal.pone.0031385 (PMC3281059; doi:10.1371/journal.pone.0031385)

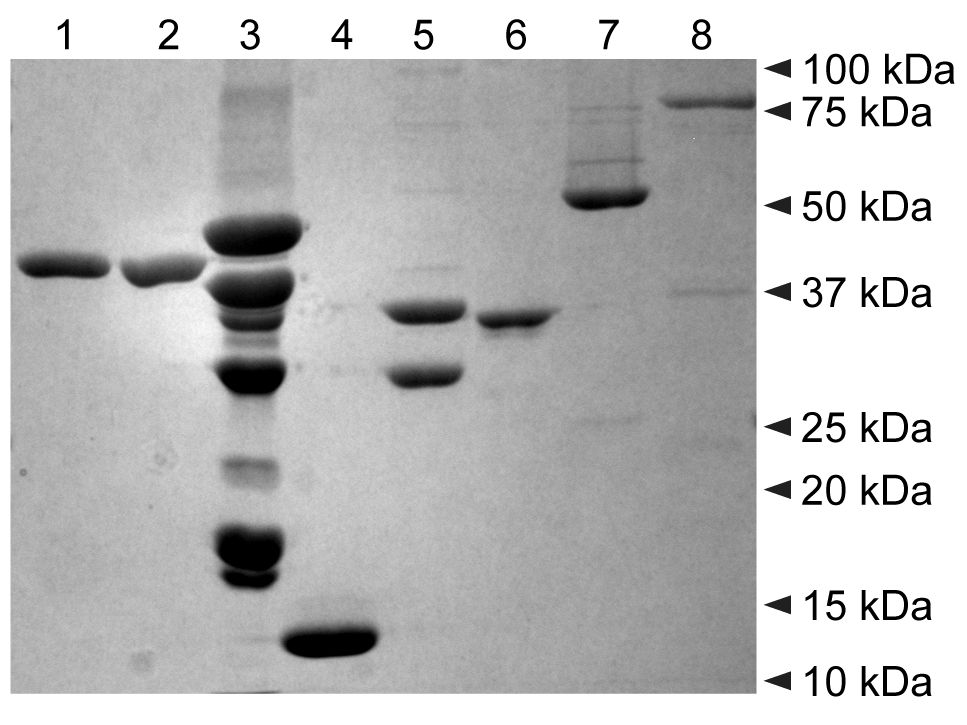

Supplement: Figure S1 — Coomassie stained SDS-PAGE gel of purified motility proteins. Lane 1, unlabeled rabbit skeletal muscle actin; Lane 2, Oregon green 488 labeled skeletal muscle actin; Lane 3, bovine thymus Arp2/3 complex; Lane 4, recombinant human profilin; Lane 5, recombinant mouse capping protein; Lane 6, recombinant glutathione sepharose transferase (GST) N-terminal tagged WCA domains from human N-WASP; Lane 7, recombinant human fascin; Lane 8; rabbit skeletal muscle myosin II heavy chain inactivated with N-Ethylmaleimide. (TIF) [file pone.0031385.s001.tif]

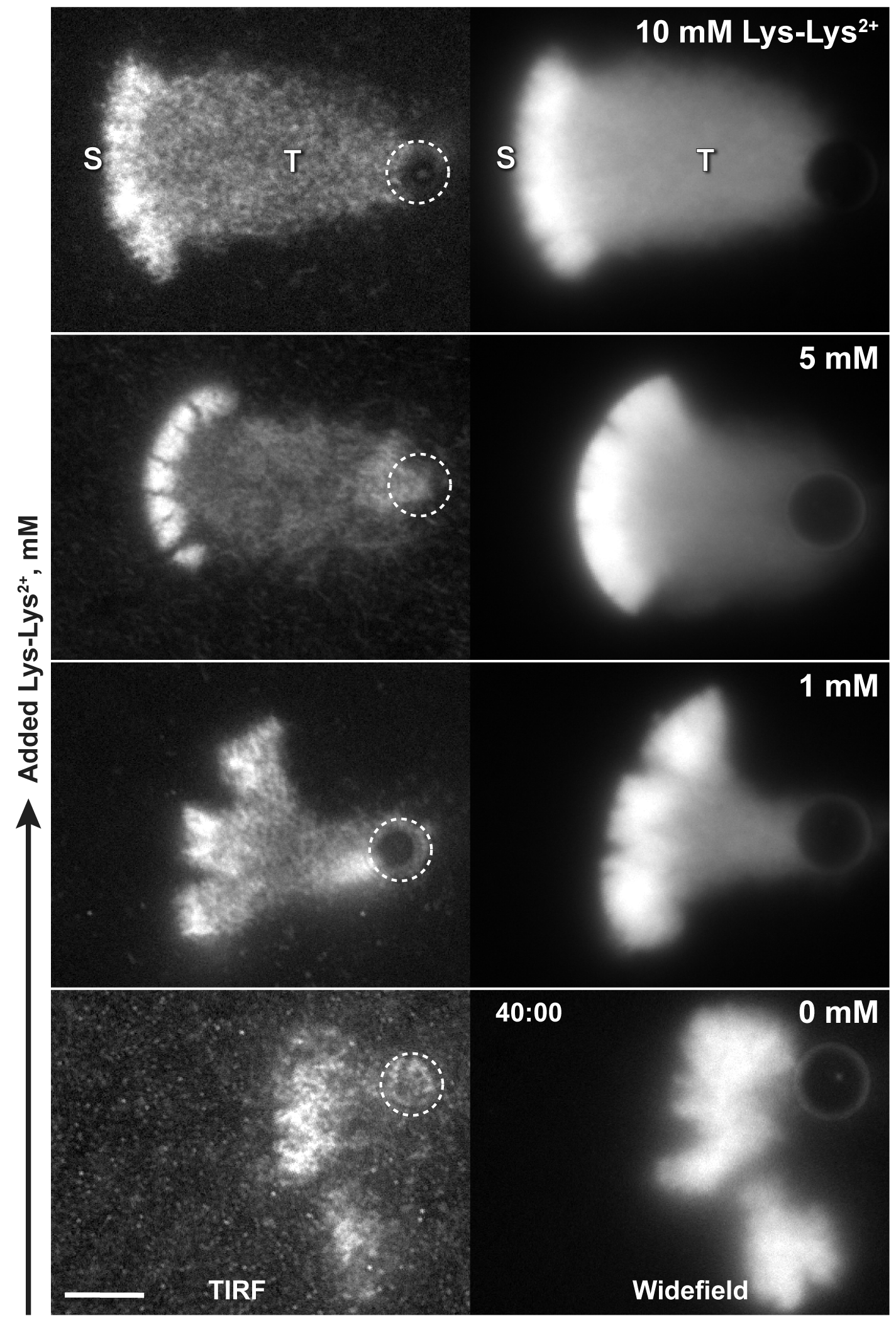

Supplement: Figure S2 — Lys-Lys2+ restores motility. Conditions as in Figure 4B. TIRF and epi-fluorescence microscopy images of actin shells (S) and comet tails (T) grown from GST-WCA coated beads in 0.1 mM total, 0.03 mM free Mg2+ buffer with added Lys-Lys2+ as indicated. Each image was recorded 40 minutes after initiation of the reaction. Lys-Lys2+ substituted for Mg2+ to restore motility. Scale bar is 5 µm. (TIF) [file pone.0031385.s002.tif]

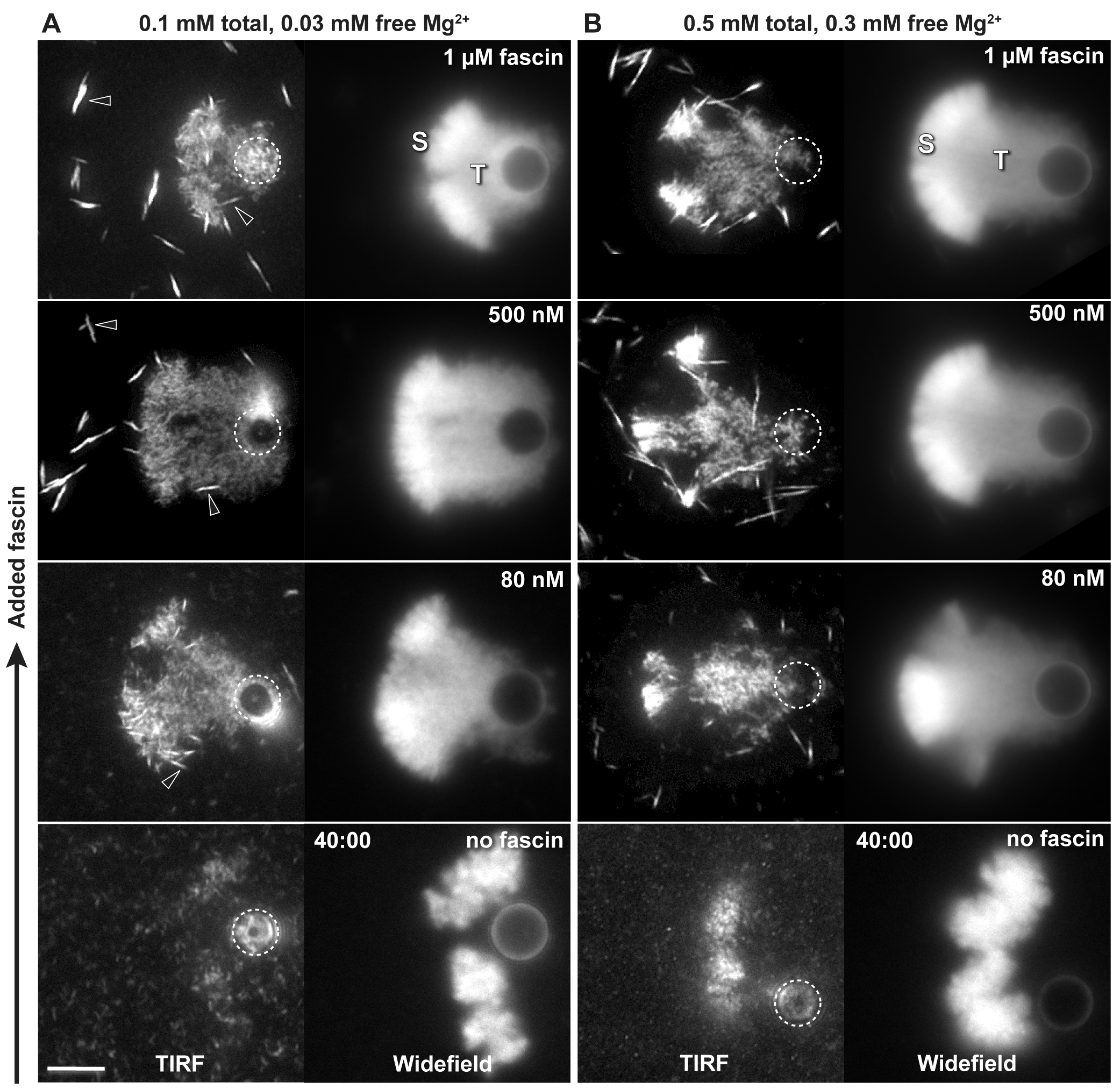

Supplement: Figure S3 — Fascin restores motility. Conditions as in Figure 4 . (A) TIRF and epi-fluorescence microscopy images of actin shells (S) and comet tails (T) grown from GST-WCA coated beads in 0.1 mM total, 0.03 mM free Mg2+ buffer with added fascin as indicated. Fascin added to 80 nM optimally restored comet tail elongation. Straight fascin bundles (black arrowheads) can be seen both within the comet tail and in the surrounding media. (B) Actin shells (S) and comet tails (T) grown in 0.5 mM total, 0.3 mM free Mg2+ buffer with added fascin as indicated. Although 0.3 mM free Mg2+ did not support motility on its own, fascin addition restored motility to a greater extent than in 0.03 mM free Mg2+. Each image was recorded 40 minutes after initiation of the reaction. Scale bar is 5 µm. (TIF) [file pone.0031385.s003.tif]

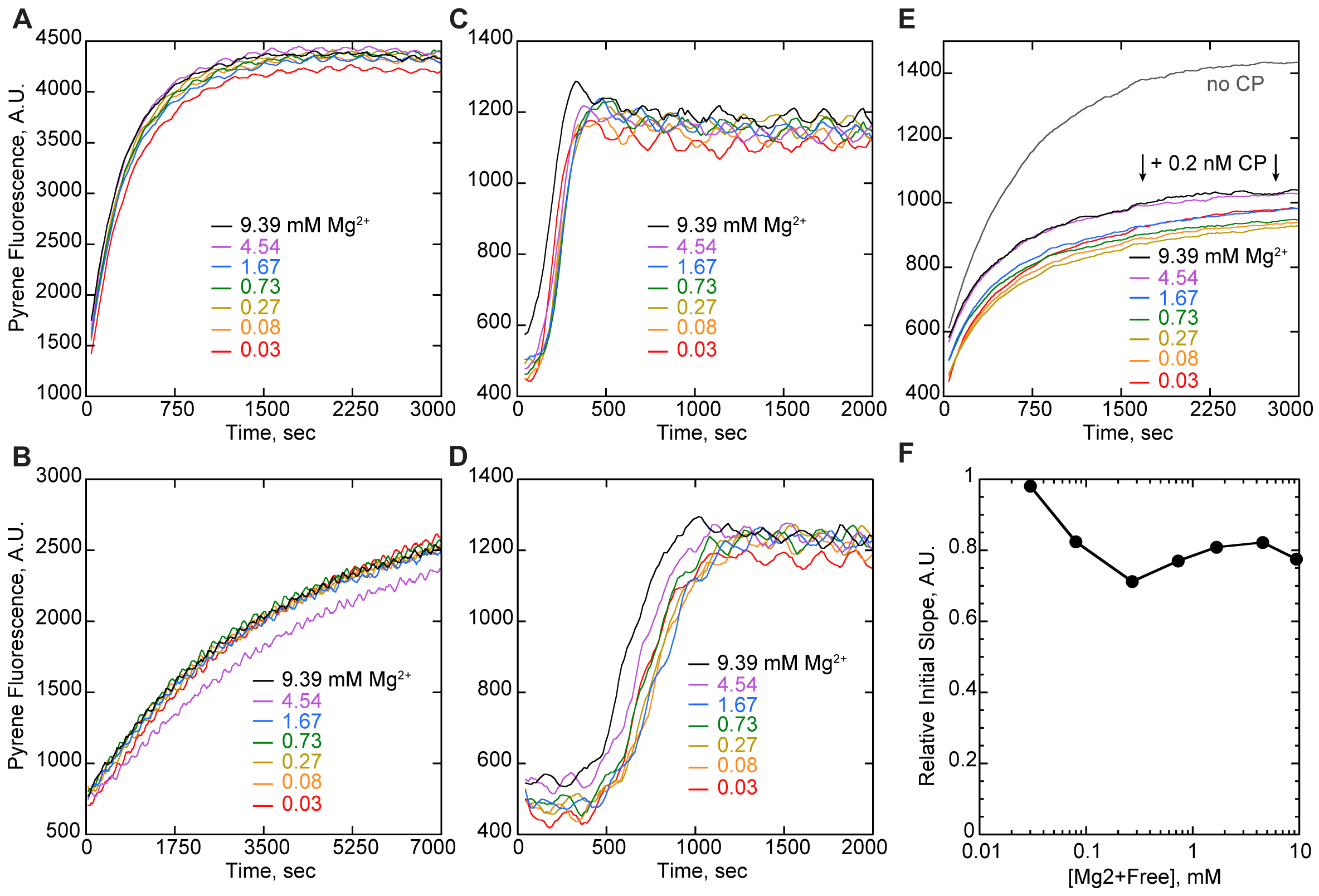

Supplement: Figure S4 — Minimal Mg2+ is sufficient for actin polymerization, Arp2/3 nucleation, and CP activity. Polymerization of pyrene actin in low Mg2+ buffer (50 mM KCl, 0.105 mM MgCl2, 1.05 mM EGTA, 10 mM imidazole pH 7.0, 0.2 mM ATP). MgCl2 was added to generate indicated free [Mg2+]. (A) Polymerization of 8.5 µM (30% pyrene labeled) Mg-ATP-actin induced by KCl was not affected by MgCl2 concentration. (B) Addition of 8.5 µM human profilin to 8.5 µM actin did not affect Mg2+ independent actin polymerization. (C) Nucleation of 2 µM (30% labeled) Mg-ATP-actin by 40 nM Arp2/3 and 500 nM bovine N-WASP WCA. Mg2+ did not affect the time course or extent of Arp2/3 mediated nucleation. (D) Nucleation conditions in C with addition of 2 µM profilin. Profilin did not significantly alter the Mg2+ independence of Arp2/3 nucleation. (E) Polymerization from capped seeds. Short unlabeled actin seeds diluted to 1.2 µM filament were incubated with 0.2 nM CP or buffer alone (no CP). Capped seeds were added to 1 µM (30% pyrene labeled) actin, 3 µM profilin at the reaction start. (F) Normalized initial slope from the first 200 s of polymerization from capped seeds in E. (TIF) [file pone.0031385.s004.tif]

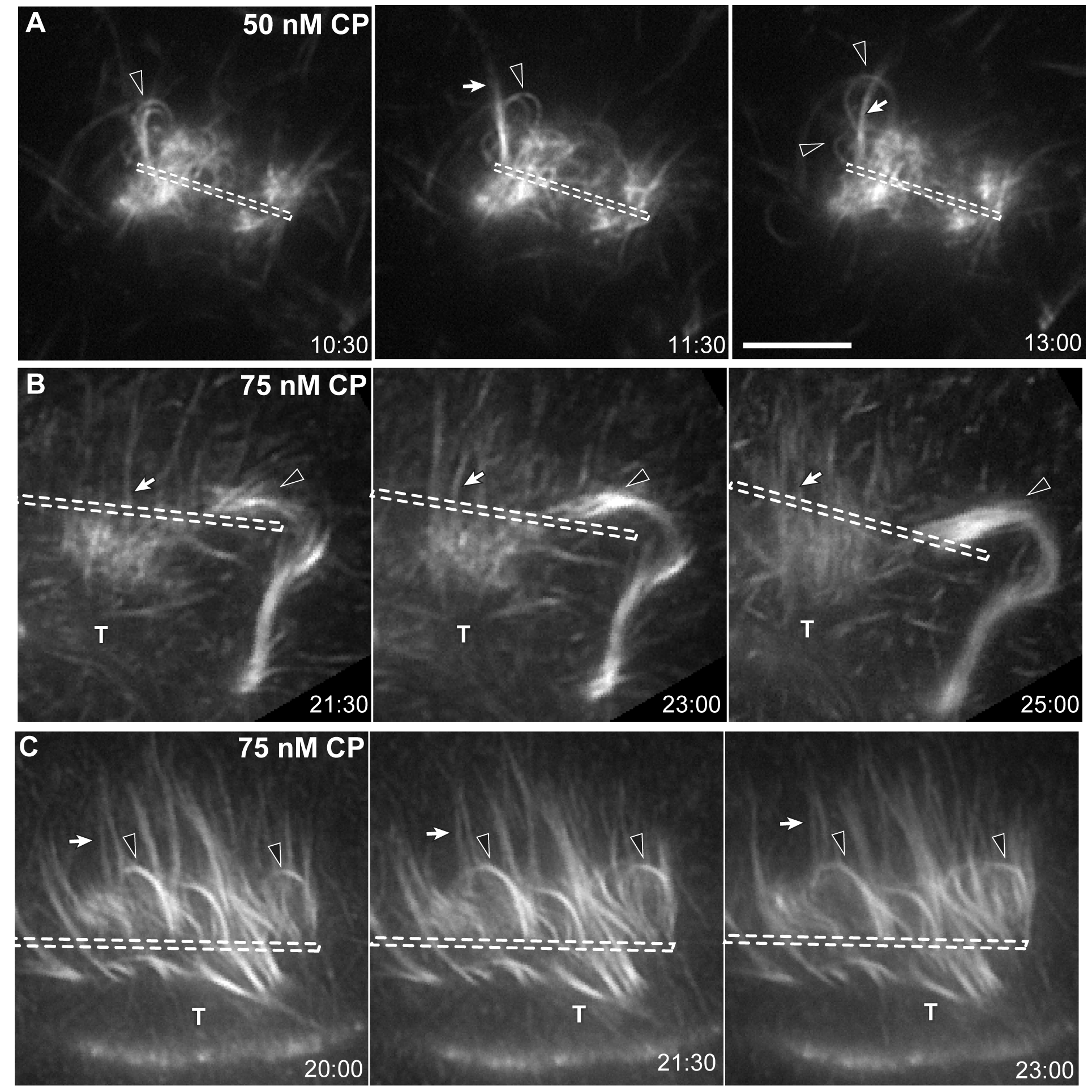

Supplement: Figure S5 — Looped bundles formed in low CP. Conditions: 8.5 µM (8% labeled) actin, 9 µM profilin, 100 nM Arp2/3, CP as indicated. (A–C) At low CP concentrations, bundled loops (black arrowheads) often formed on both stationary (A) and moving (B–C) nanofibers. Loops grew with one end embedded in the comet tail (T) and the other attached to the nanofiber surface (dashed outline). In additional to looped bundles, straight bundles (white arrows) often projected beyond the nanofiber surface at low CP concentrations. Scale bar, 5 µm. Time, min: sec. (TIF) [file pone.0031385.s005.tif]

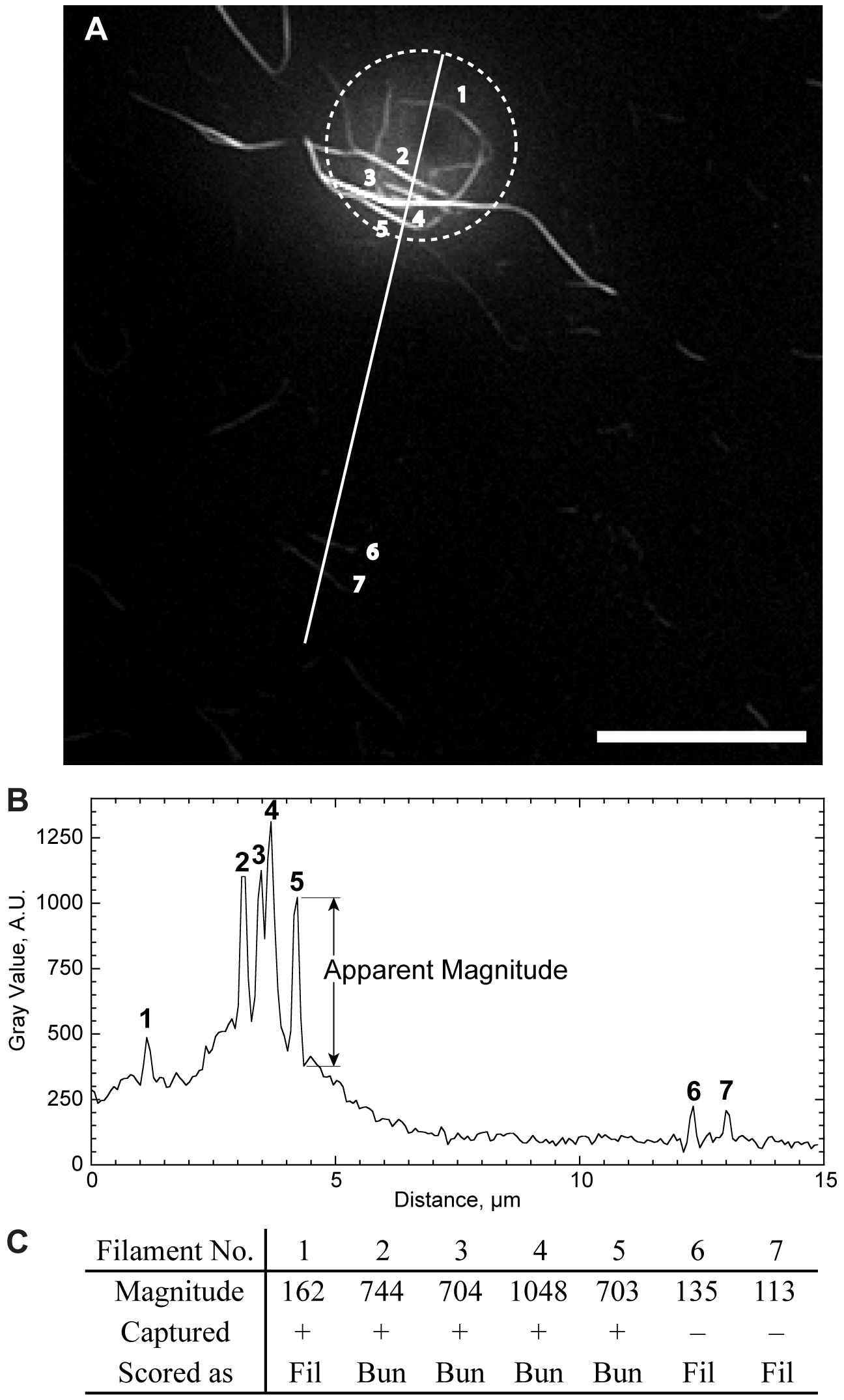

Supplement: Figure S6 — Scoring of bundles captured by GST-WCA coated beads. (A) Sample TIRF microscopy image of actin filaments captured by a GST-WCA coated bead (dashed circle) in 0.03 mM free Mg2+ supplemented with 1 mM Lys-Lys2+. Scale bar, 5 µm. (B) Profile plot of fluorescent intensity along the line in A that intersects seven filaments. Numbered peaks correspond to marked filaments. Two dim background filaments are included for comparison. (C) Sample scoring method for experiments shown in Figure 7 . Camera gain, acquisition time, and display range were kept constant between experiments to give roughly the same apparent magnitude (peak-to-trough intensity) of background filaments (6–7). Filaments were scored as captured (+) if they crossed or were contained within the bead boundary as measured with DIC microscopy. Captured filaments with apparent magnitudes similar to background filaments were scored as individual filaments (Fil). Captured filaments with apparent magnitudes of at least double the average magnitude of background filaments were scored as bundles (Bun). (TIF) [file pone.0031385.s006.tif]
